# Comprehensive Analysis of Carotenoid Cleavage Dioxygenases Gene Family and Its Expression in Response to Abiotic Stress in Poplar

**DOI:** 10.3390/ijms23031418

**Published:** 2022-01-26

**Authors:** Hui Wei, Ali Movahedi, Guoyuan Liu, Yixin Li, Shiwei Liu, Chunmei Yu, Yanhong Chen, Fei Zhong, Jian Zhang

**Affiliations:** 1 Key Laboratory of Landscape Plant Genetics and Breeding, School of Life Sciences, Nantong University, Nantong 226000, China; 15850682752@163.com (H.W.); cjqm1989@126.com (G.L.); liyixinlyx0@163.com (Y.L.); 2009110160@stmail.ntu.edu.cn (S.L.); ychmei@ntu.edu.cn (C.Y.); chenyh@ntu.edu.cn (Y.C.); fzhong@ntu.edu.cn (F.Z.); 2 Key Lab of Landscape Plant Genetics and Breeding, Nantong 226000, China; 3 Co-Innovation Center for Sustainable Forestry in Southern China, Key Laboratory of Forest Genetics & Biotechnology, Ministry of Education, College of Biology and the Environment, Nanjing Forestry University, Nanjing 210037, China; ali_movahedi@njfu.edu.cn; 4 College of Arts and Sciences, Arlington International University, Wilmington, DE 19804, USA

**Keywords:** carotenoid, CCD, NCED, ABA, poplar

## Abstract

Carotenoid cleavage dioxygenases (CCDs) catalyzes the cleavage of various carotenoids into smaller apocarotenoids which are essential for plant growth and development and response to abiotic stresses. CCD family is divided into two subfamilies: 9-cis epoxycarotenoid dioxygenases (NCED) family and CCD family. A better knowledge of carotenoid biosynthesis and degradation could be useful for regulating carotenoid contents. Here, 23 *CCD* genes were identified from the *Populus trichocarpa* genome, and their characterizations and expression profiling were validated. The PtCCD members were divided into PtCCD and PtNCED subfamilies. The PtCCD family contained the PtCCD1, 4, 7, and 8 classes. The PtCCDs clustered in the same clade shared similar intron/exon structures and motif compositions and distributions. In addition, the tandem and segmental duplications resulted in the *PtCCD* gene expansion based on the collinearity analysis. An additional integrated collinearity analysis among poplar, Arabidopsis, rice, and willow revealed the gene pairs between poplar and willow more than that between poplar and rice. Identifying tissue-special expression patterns indicated that *PtCCD* genes display different expression patterns in leaves, stems, and roots. Abscisic acid (ABA) treatment and abiotic stress suggested that many *PtCCD* genes are responsive to osmotic stress regarding the comprehensive regulation networks. The genome-wide identification of *PtCCD* genes may provide the foundation for further exploring the putative regulation mechanism on osmotic stress and benefit poplar molecular breeding.

## 1. Introduction

Terpenoids consist of various primary and secondary metabolites that function in all living organisms. Carotenoids and their apocarotenoids, considered C_40_ isoprenoids, participate in multiple essential biological functions in plants and animals [1,2]. For example, xanthophylls and violaxanthin are the critical components of plant light-harvesting protein complexes [3], and carotenoids in chloroplasts are involved in light absorption, electron transfer, and removal of triplet oxygen and superoxide anion in plant photosynthesis [4,5]. In addition, lycopene and β-carotene are important pigments that affect the color of plant flowers and fruits, and their contents are associated with fruit colors and qualities [6,7]. In addition, zeaxanthin aldehyde, a carotenoid derivative, can be catalyzed into abscisic acid (ABA). ABA, also known as a stress hormone, plays a vital role in regulating various physiological and developmental processes. It can not only act as a growth inhibitor to intervene in biological processes such as plant flowering [8], fruit maturation [9], and seed dormancy [10], but also directly responds to drought, salt, and low temperature through the ABA signal transduction pathway including mediation of stress-resistant genes expression [11], reducing transpiration [12,13], and inducing stomatal closure [14,15].

Carotenoid cleavage dioxygenases (CCDs) are mainly responsible for the oxidative cleavage of carotenoids in higher plants, which results in the biosynthesis of biologically smaller apocarotenoids [5]. CCDs are also a kind of non-heme iron dioxygenase, which contain the RPE65 (retinal segment epithelial membrane protein) domain responsible for binding Fe^2+^ [16,17]. In higher plants, the carotenoid cleavage dioxygenase (CCD) and 9-cis carotenoid cleavage dioxygenase (NCED) have been identified as a subfamily of CCDs based on the epoxy structure of substrate [18,19]. NCEDs can catalyze the cleavage of 11, 12 double bond of violaxanthin (C_40_) or neoxanthin (C_40_) to form the xanthoxin (C_15_), and this catalytic reaction carried out by NCEDs is considered as the rate-limiting step in ABA biosynthesis [20,21]. A variety of studies have been focused on the regulation mechanism of NCEDs in resistance to stress conditions. In Arabidopsis, NCED members were classified into NCED2, 3, 5, 6, and 9 according to phylogeny and gene function [18]. The transcript levels of *AtNCED3* could be induced by drought stress, and AtNCED3 controls the level of endogenous ABA. Overexpression of *AtNCED3* could improve drought tolerance of Arabidopsis by reducing leaf transpiration rates [22]. AtNCED6 and AtNCED9 were associated with developmental control of ABA biosynthesis in seeds. In addition, both AtNCED2, AtNCED3, and AtNCED5 have essential roles in endogenous ABA accumulation [23]. In rice, OsNCED members have also been classified into OsNCED1, OsNCED2, OsNCED1, OsNCED4, and OsNCED5, and the divergent expression of *OsNCED* members in tissues resulted in different functions [24]. OsNCED1 and OsNCED3 were involved in the drought resistance, and OsNCED2 was associated with the delay of seed germination [25]. Overexpression of *OsNCED5* and *OsNCED3* in Arabidopsis increased the tolerance to drought stress and delayed seed dormancy and changed plant size and leaf morphology [26,27]. *CsNCED* genes from *Crocus sativus* had a closer relationship with ABA accumulation under the drought, salt, and lower temperature [28]. These results suggested that NCEDs control ABA biosynthesis and then affect the ABA-mediated signal transduction pathway involved in plant stress responses.

In contrast, CCD enzymes do not have the specific cleavage sites of substrates. Some CCDs can cleave carotenoid or apocarotenoid substrates, while others recognize specific carotenoid or apocarotenoid substrates [29]. CCD subfamily of Arabidopsis was divided into CCD1, CCD4, CCD7, and CCD8 [17]. AtCCD1 and AtCCD4 mainly cleaved 9, 10 double bonds leading to catalyze the C_40_ carotenoids and C_27_ terpenoids to form the small molecule volatile substances, such as C_13_ β-ionone and C_14_ geranyl acetone geranyl acetone, which suggested that both AtCCD1 and AtCCD4 have an important influence on the formation of plant aroma [30,31]. AtCCD7 catalyzed β-carotene to form β-ionone and C27 10′-apo-β-carotenal, and AtCCD8 or AtCCD1 could further cleave C_27_ 10′-apo-β-carotenal to generate C_18_ 3′-apo-β-carotenal or β-ionone and one apo-10,10′-carotendial, respectively [32,33]. The observations illustrated that AtCCD7 and AtCCD8 could oxidize β-carotene to produce the strigolactone (SL) precursor related to the important biological processes such as branch formation, lateral root formation, seed germination, and response to drought and salt stresses [34]. The composition of CCD4 members is complex, and only one CCD4 member has been identified in Arabidopsis, while many CCD4 members have been identified in tomato, grape, and saffron [35,36,37]. The evidences on CCD4 members show that CCD4 members may be involved in forming a flower, peel, and pulp color. The β-cryptoxanthin and zeaxanthin, thought as the substrate of citrus CCD4b1, are cleaved at the 7, 8 double bonds to form unique C_30_ carotenoid associated with the color of orange peel [38]. In addition, the expression level of *Glycine max CCDs* has been significantly changed under salt, drought, low temperature, and high-temperature stresses, indicating that soybean CCDs are involved in its abiotic stress response process [39]. *Brassica oleracea* CCD1 and CCD4 are responsive to drought and salt stresses [40]. The expressions of *Malus domestica CCDs* are significantly affected under salt and drought stress, indicating that MdCCD members are involved in response to the abiotic stresses [41]. Given the above evidence, CCD members have various kinds of biological functions and participant in regulating plant growth and development, abiotic stresses, and color formation. However, so far, there is no genome-wide identification of CCD family genes in poplar.

Studies on genome-wide identification of gene families have focused on their characterizations and functions and provided valuable methods for analyzing gene networks or biological functions. For example, analysis of pin-formed (PIN) gene family in wheat displayed PIN may be involved in various developmental processes and biotic and abiotic stress conditions [42]. Identification of β-ketoacyl CoA synthetase (KCS) in barley exhibited barley KCS members function in regulating physiological and biochemical processes and participant in drought stress [43]. In addition, genome-wide identification and characterization of lncRNAs in *Capsicum annuum* showed that lncRNAs interaction with miRNAs takes part in different abiotic stress by regulating transcription factors (TFs), including tryptophan arginine lysine tyrosine (WRKY), myeloblastosis (MYB), basic leucine zipper domain (bZIP), and so on [44]. In addition, genome-wide analysis of the wheat brassinazole-resistant (BZR) gene family exhibited that BZRs play crucial roles in plant developmental processes and are associated with diverse biotic and abiotic stresses [45]. Genome-wide identification of *Salvia miltiorrhiza* antisense transcripts (NATs) considered as a class of long noncoding RNAs documented that NATs interaction with sense transcripts (STs) potentially plays significant regulatory roles in the biosynthesis of bioactive compounds [46]. Genome-wide investigation of the soybean AT-hook motif nuclear localized (AHL) gene family proved that AHLs mainly react to mediating stress responses [47].

Moreover, the identification of tobacco CCD family revealed that tobacco CCD genes have essential roles in response to different hormones, including ABA, methyl jasmonate (MeJA), indoleacetic acid (IAA), salicylic acid (SA), and abiotic stresses [48]. The above genome-wide identification provides an essential theoretical basis for understanding family gene physiological and biochemical functions in different species. Poplar and willow mainly distributed in the cold zone to the temperate zone of the northern hemisphere belong to *Salicaceae Mirb* [49,50]. Poplar is also a model plant to study the molecular mechanism of growth and development, material properties, stress responses, and other vital traits. In addition to being used as an energy tree for industrial production, poplar can also protect the soil structure, prevent and control soil erosion, and have a significant ecological value. In recent years, with the destruction of the ecological environment and the drastic change of the global climate, the inevitable natural disasters such as drought, salt, and freezing have seriously restricted the natural growth and development of poplar and have caused a decline in poplar production capacity and seriously damaged the balance of the local ecological environment. Considering the physiological functions of CCD members under the various stresses, we identify CCD family members of *Populus trichocarpa* at the whole genome level. We systematically analyze molecular evolution, gene structure, cis-acting elements, and conserved motifs of PtCCD family members. In addition, we evaluate the transcript levels of *PtCCDs* in various tissues and identify the expression patterns of *PtCCDs* under abiotic stress. The above results will lay a foundation for further elucidating the biological functions of PtCCD family members.

## 2. Results

### 2.1. Characterization of the CCD Family Members in Poplar

The 30 candidate PtCCD members were obtained from the genome database of *P. trichocarpa* (Phytozome https://phytozome-next.jgi.doe.gov/pz/portal.html (accessed on 28 July 2021)) based on sequence alignment with Arabidopsis CCD members (Table S1). The complete RPE65 domain was used as the standard for screening the above PtCCD members and four members (Potri.006G238500, Potri.006G239301, Potri.018G043201, Potri.018G044100) displayed no dominant RPE65 domain were eliminated from the PtCCD members. In addition, Potri.T074400 and Potri.T167700 remained on scaffolds, which were not spliced with the poplar chromosome, so Potri.T074400 and Potri.T167700 were abandoned for analysis. Finally, 23 PtCCD members possessing complete RPE65 domain were retrieved from the genome of poplar (Table 1). The PtCCD members were named based on their orthologous relationship with Arabidopsis and rice CCD family members (Table 1). Sequence alignment illustrated significant differences in PtCCD sequences, but the four histidines (His) sites are highly conserved (Figure S1).

The open reading from (ORF) lengths ranged from 1221 to 1842, except for the incomplete sequence information on Potri.004G190700, Potri.009G152300, Potri.018G042900, and Potri.018G043400. The deduced amino acids of PtCCDs varied from 406 to 613, and the molecular weights (MWs) ranged from 46.28–69.07 KD. A high proportion of PtCCDs theoretical PI was less than 7, indicating that a large portion of them belongs to acidic protein, except PtCCD4a, 4c, 4d, 4e, PtCCD7, and PtNCED2. In addition, the grand average of hydropathicities of PtCCDs was less than 1, which illustrated that PtCCDs are hydrophilic and non-transmembrane proteins. The analysis of instability index showed that PtCCD4c, 4d, 4e, 4f, PtCCD8d, 8e 8f, 8g, 8h, and PtNCED2, 3a, and 3b belong to unstable proteins, while others belong to stable proteins. The signal peptide prediction showed that only PtCCD8a has a classical secretion signal peptide, and the cleavage site is located in positions 16 and 17. Other PtCCD proteins do not have signal peptides. In addition, subcellular localization prediction showed PtCCDs are distributed in chloroplast and cytoplasm.

### 2.2. Phylogenetic Analysis of PtCCD Family

**Figure 1:**
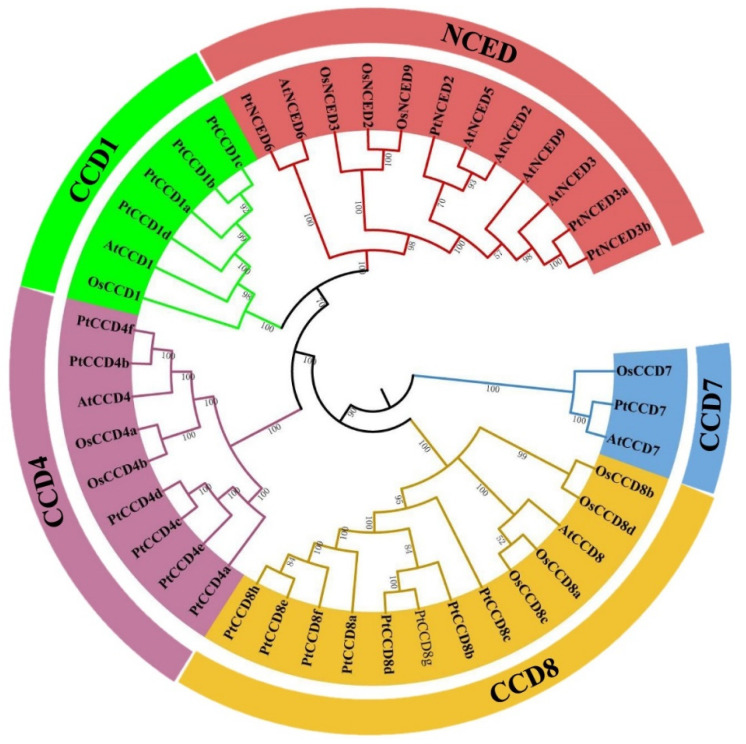
Phylogenetic tree displaying the evolutionary relationships among poplar, Arabidopsis, and rice CCDs. The neighbor-joining (NJ) method tree with 1000 bootstrap replicates was applied to draw a phylogenetic tree with the MEGA7 software. The CCD from poplar, Arabidopsis, and rice were involved in CCD and NCED families, and the CCD family were clustered into CCD1, 4, 7, and 8 classes.

To better understand the evolutionary relationships among CCD members, 9 Arabidopsis, 11 rice, and 23 poplar CCD members were chosen to construct a phylogenetic tree. According to the evolutionary relationship of Arabidopsis CCDs and rice CCDs, PtCCD family members were divided into two families and named PtCCD subfamily (PtCCD1, PtCCD4, PtCCD7, and PtCCD8 classes) and PtNCED subfamily (Figure 1). Based on clustering analysis, four CCD members were clustered into the CCD1 class, whereas only one AtCCD1 or OsCCD1 was fell into the CCD1 class. In addition, 6 PtCCD4, 1 AtCCD4, and 2 OsCCD4 members were classified into CCD4 class. In addition, the CCD8 class contained eight members in poplar, four in rice, and only one in Arabidopsis, suggesting that CCD8 members were probably expanded only within some species.

Moreover, NCED5 and NCED9 classes were not found in poplar, but two members are identified as PtNCED3 class. Furthermore, the phylogenetic tree revealed that CCD4 and CCD8 classes have a closer evolutionary relationship, and CCD1 class and NCED subfamily were clustered together, indicating that the NCED type may be evolved from the CCD1 class. The CCD1, CCD4, CCD7, CCD8, and NCED among Arabidopsis, rice, and poplar were assembled in the evolutionary clade, suggesting that the plant CCD gene family is relatively more conservative among different species, and CCD evolution is later than that of herbs and woody plants, monocotyledons, and dicotyledons.

### 2.3. Gene Structures and Conserved Motifs of CCD Members

**Figure 2:**
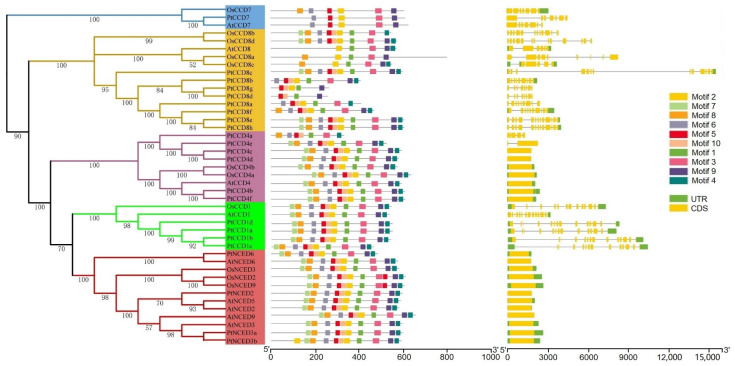
The poplar, Arabidopsis, and rice *CCD* gene structures and conserved motifs. Exons and introns were represented using bars and rectangles, respectively. The conserved motifs were displayed using colorful rectangles.

To better understand the relationship among the phylogenetic evolution, gene structures, and the conserved motifs of CCD family members, the exon/intron structures and distributions of conserved motifs together with the phylogenetic tree of the CCD family were analyzed. As expected, the *CCD* members possessed similar exons/introns were clustered into the same clade. All *PtNCED* members had no intron, and similar gene structures were identified in *PtCCD4* (except *PtCCD4a* and *PtCCD4e*). Furthermore, the *PtCCD1*, *7*, and *8* had many introns and relatively comprehensive exon/intron structures compared with *PtNCED* and *PtCCD4* (Figure S2). Obviously, in addition to *PtCCD* gene structures, the *AtCCD* and *OsCCD* genes shared similar exon/intron structures (Figure 2).

To explore the features of CCD member motifs, ten distinct motifs were chosen to investigate using the MEME tool. Interestingly, motif distributions of CCDs were similar in the same clade. The CCD1, CCD4, and NCED proteins contained motifs 1–10, suggesting that motifs 1–10 might be involved in their standard functions. In addition, the motif compositions of CCD8 members were divergent, which illustrated that CCD8 members may participate in various physiological processes and could be responsible for different functions. In addition, the number of motifs in CCD7s was lower than that in CCD1, CCD4, and NCED members, which illustrated that CCD7s are speculated to be lacking some specific biological functions compared with CCD1, CCD4, and NCED members (Figure 2). In conclusion, these observations suggested different conserved motifs and gene structures in CCD classes, which further supported the CCD family phylogenetic clustering.

### 2.4. Expansion and Contraction of *PtCCD* Genes

**Figure 3:**
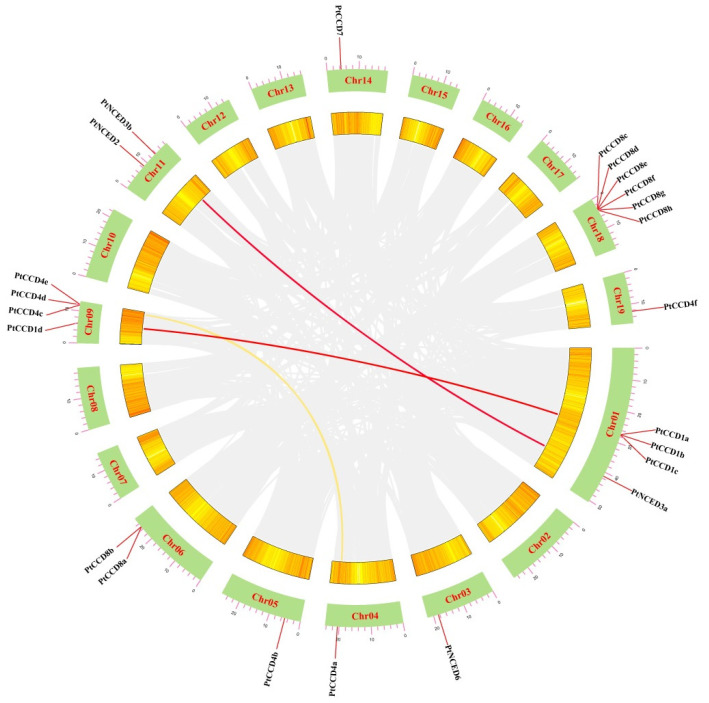
The collinearity analysis of *PtCCDs*. Chromosomes 01–19 were indicated using green rectangles. The homologous *PtCCD* genes in the poplar genome were displayed using red and yellow curves. The poplar gene with collinearity relationship was represented using gray curves.

The chromosomal distributions of *PtCCD*s were determined and visualized based on the TBtools and poplar genome annotation information. In general, 23 *PtCCD* members were unevenly anchored to Chr01, Chr03–06, Chr09, Chr11, Chr14, Chr18, and Chr19 (Supplemental Figure S3). Chr01, as the largest chromosome, contained 4 *PtCCD* genes, and 6 *PtCCD* genes were mapped to Chr08, while Chr03–05, both 14 and 19, contained only one *PtCCD* gene, respectively. The method of gene family formation usually contains tandem, segmental, and whole-genome duplications. Gene duplication analysis might provide helpful information for *CCD* family gene formation. Here, tandem duplication and segmental duplication are evaluated by the multiple collinearity scan toolkit (MCScanX) method. In general, the tandem duplication origin from homologous genes located on the same chromosome. The gene pairs, including *PtCCD4d* and *PtCCD4e*, and *PtCCD8e* and *PtCCD8f* thought as tandem duplication events were identified in the *PtCCD* gene family. Meanwhile, the *PtCCD4d* and *PtCCD4e*, and *PtCCD8e* and *PtCCD8f* shared similar gene structures and motif distributions. In addition, homologous genes on the different chromosomes were probably the result of segmental duplication. Collinearity analysis of the *PtCCDs* showed that *PtCCD1a* and *PtCCD1d*, *PtCCD4a* and *PtCCD4c*, and *PtNCED3a* and *PtNCED3b* have collinearity relationships, respectively (Figure 3). The *PtCCD1a* and *PtCCD1d*, or *PtNCED3a* and *PtNCED3b* share 91.65% or 91.67% similarity, respectively, which illustrated that gene pairs are formed by segmental duplication. The above observations indicated that expansion of *PtCCD* genes might probably be original from both tandem duplication and segmental duplication. Moreover, *PtCCD8c*, *8d*, *8e*, *8f*, *8g*, and *8h* are located on chromosome 18, *PtCCD1a*, *1b*, and *1c* located on chromosome 1 *PtCCD4c*, *4d*, and *4e* located on chromosome 9 formed various gene clusters. Furthermore, to clarify the role of selection pressure in the evolution of the *PtCCD* genes, TBtools were used to analyze the Ks, Ka, and Ka/Ks of homologous *PtCCD* genes. The results suggested that Ka/Ks values in 2 pairs of tandem duplication and 3 pairs of genome duplication are less than 1, indicating that the *PtCCD* family has undergone strong purification selection during the evolution process.

**Figure 4:**
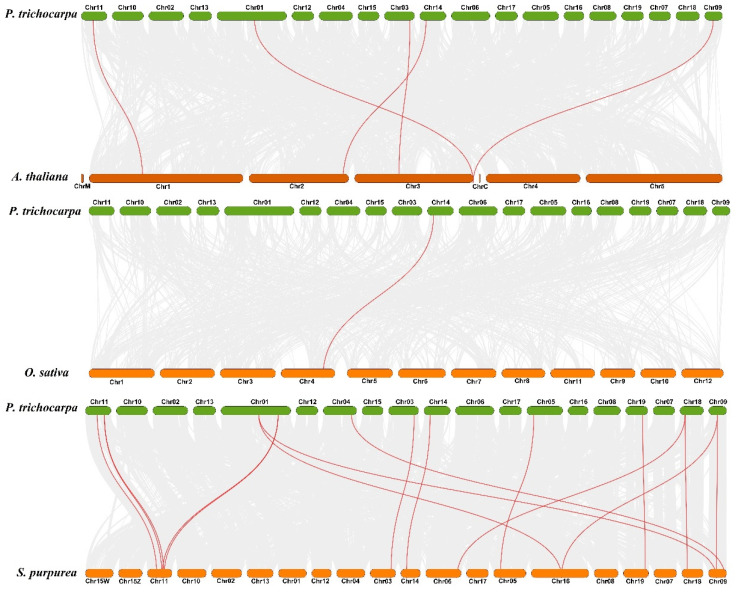
Analysis of collinearity among poplar, Arabidopsis, rice, and willow *CCD* genes. All putative orthologous genes were represented using gray curves, and the orthologous *CCD* genes among the genomes of poplar, Arabidopsis, rice, and willow were displayed using red curves.

To explore the orthologous relationship among poplar, Arabidopsis, rice, and willow, the analysis of collinearity relationship was performed using TBtools with MCScanX. The result showed that 5 gene pairs are found between poplar and Arabidopsis, 1 gene pair is found between poplar and rice, and 16 gene pairs are found in poplar and willow, respectively (Figure 4). In the evolutionary relationship of species, poplar and willow were classified to Salicaceae Mirb, and poplar and Arabidopsis were classified to dicotyledonous plants. The collinearity relationship among poplar, Arabidopsis, rice, and willow suggested that poplar has a closer evolutionary relationship with willow.

### 2.5. Three-Dimensional (3D) Structures and Cis-Acting Elements Analysis

The results of 3D structure prediction showed that CCD proteins consist of coils, strands, and helixes (Supplemental Figure S3). The coils and strands occupied most CCD structures, while helixes only accounted for a small part of CCD structures. The same class, including CCD1, 4, and 7 and NCED, was visualized to possess the relatively higher similarity, and the divergences lay mainly in the coils and helixes. The 3D structures of CCD8 members were relatively comprehensive and various. The 3D structures of PtCCD8c, PtCCD8d, and PtCCD8g were dominantly divided into two parts marked A and B (Supplemental Figure S4), and the two parts of 3D structures were connected by coils or helixes.

In comparison, the other CCD8 members have no two significant parts of 3D structures. Based on the comparative analysis of CCD8 structures, nearly all 3D structures of CCD8 members could be highly merged in structure A, suggesting that structure A in CCD8 members may be relatively conservative compared to structure B. In addition, the CCD1 members had similar 3D structures with NCED members, which might explain the closer evolutionary relationship between CCD1 and NCED members in a phylogenetic tree. 

**Figure 5:**
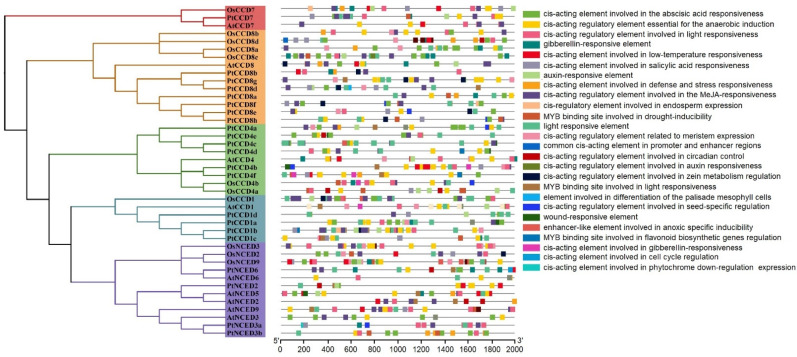
Analysis of poplar and Arabidopsis *CCD* gene promoter cis-elements. Left panel, the phylogenetic tree constructed by NJ method. Right panel, the kinds of *CCD* promoter cis-elements displayed using colorful rectangles.

To understand the putative biological functions of CCD members, PlantCARE was used to analyze the cis-acting elements in the promoters of *CCDs*. The results showed that the *CCD* promoters contain various cis-acting elements, divided into two categories. One is abiotic stress response elements, such as anaerobic inducible element (ARE), stress response element (STRE), MYB drought inducible binding site (MBS), DREB/CBF transcription factor recognition site (DRE), disease resistance, and stress-inducing elements (TC-rich repeats) and low-temperature response elements (LTR). The other category is plant hormone response elements, such as auxin response elements (TGA-element), salicylic acid (SA) inducing elements (TCA-element), methyl jasmonate (MeJA) response element (CGTCA-motif), and ABA response element (ABRE). In addition, the number of light-responsive elements, anaerobic inducing elements, and ABA-responsive elements occupied a large part of cis-acting elements in the *PtCCD* and *AtCCD* promoters, and *OsCCD* promoters contained large numbers of light-responsive elements, MeJA-responsiveness elements, and ABA-responsive elements. Both of *PtCCD* promoters, *PtCCD1b*, *PtCCD4d*, *PtCCD7,* and *PtCCD8f* had a large proportion of MeJA-responsive elements in their promoters, and *PtCCD4b* and *PtNCED2* contained relatively some low-temperature responsive elements. In addition, ABA-responsive elements were widely distributed in *PtCCD4e*, *PtNCED3b*, *PtCCD1a*, *PtNCED3a*, and *PtCCD4e* promoters, while *PtCCD8a*, *PtCCD8b*, and *PtCCD8e* contained fewer cis-acting elements (Supplemental Figure S5). For *AtCCD* promoters, ABA-responsive elements were widely present in *AtNCED5*, *AtNCED9,* and *AtNCED3* promoters. MYB binding sites involved in drought-inducibility existed in *AtNCED9*, *AtCCD4*, *AtCCD8*, *AtNCED3,* and *AtNCED6*. For analysis of *OsCCD* promoters, *OsCCD8c*, *OsCCD8a*, *OsNCED9*, *OsNCED3*, and *OsCCD8d* contained large numbers of ABA-responsive elements. *OsCCD8c*, *OsCCD7*, *OsNCED3*, *OsCCD8b*, *OsCCD4b*, *OsNCED2*, and *OsCCD1* contained the certain number of MeJA-responsive elements. MYB binding sites involved in drought-inducibility were predicted in *OsCCD4a*, *OsCCD4b*, *OsCCD8c*, *OsNCED2*, and *OsNCED9* promoters. Overall, the analysis of cis-acting elements in *CCD* promotes illustrated that *CCDs* transcript levels may be regulated by light, hormone, and abiotic stress, and CCDs were speculated to be involved in diverse stress resistances (Figure 5).

To analyze the influence of CCDs evolution on their promoter elements, *CCD* cis-acting elements and CCDs phylogenetic tree were compared. The results showed a minor difference in *PtCCDs* cis-acting elements in the same clade. The compositions and distributions of *PtCCDs* cis-acting elements in different evolutionary clades have significant differences. In addition, the other species’ *CCD* cis-acting elements in the same clades showed substantial differences, suggesting that orthologous CCDs within the different species may exist the functional differentiation. All above evidence indicated that the categories of *CCD* cis-acting elements are different with CCD evolutionary relationship.

### 2.6. Interaction Networks of Protein-Protein Assays

Interaction network analysis can find the relationship of protein–protein. These proteins may be mutually regulated, tightly related in function, or members involved in the same signaling pathway or physiological process. Here, the String database (https://string-db.org/ (accessed on 28 July 2021)) and Cytoscape software were used to identify the interaction network. As shown in Supplemental Figure S6, AtCCD7 was located at the core position in interaction network of AtCCD family members. In addition, the interaction network in PtCCD family members was more sophisticated than that in AtCCD family members. In interaction network of PtCCD family members, PtCCD8g, PtCCD7, and PtCCD4a were predicted to interact together and were in the core position. The above results predicted that PtCCD8g, PtCCD7, and PtCCD4a interaction with other PtCCDs functions together and participants in the same signal transduction and biological process.

**Figure 6:**
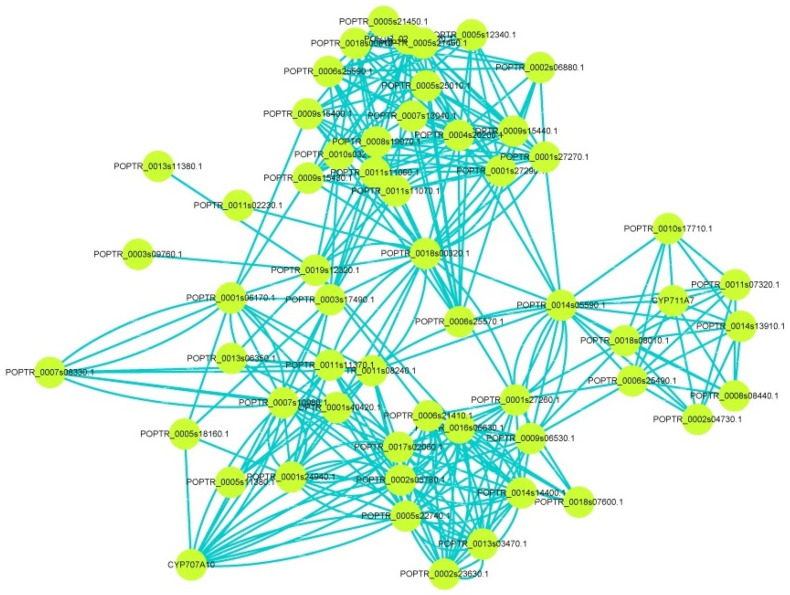
The interaction network for PtCCD members in poplar. The String database (https://string-db.org/ (accessed on 28 July 2021)) was used to predict the interaction relationship between PtCCD members and other proteins, and Cytoscape software was applied to visualize the interaction network.

Moreover, to further discover the functions of CCDs, interaction assays were performed to construct the relationship between CCD members and other proteins. The result showed that CCDs might interact with cytochrome P (CYP), phytoene synthase (PSY), abscisic acid insensitive (ABI), gibberellic oxidase (GAox), thaumatin-like protein (TCP), and so on, implying CCDs might exert functions by interacting with other genes (Figure 6 and Supplemental Figure S7). The above results predicted that PtCCDs might play an essential role in ABA and GA biosynthesis or ABA and GA signal transduction. Overall, interaction networks could provide crucial references for identifying the regulation mechanism of PtCCDs.

### 2.7. Expression Patterns of *PtCCDs* in Different Tissues

**Figure 7:**
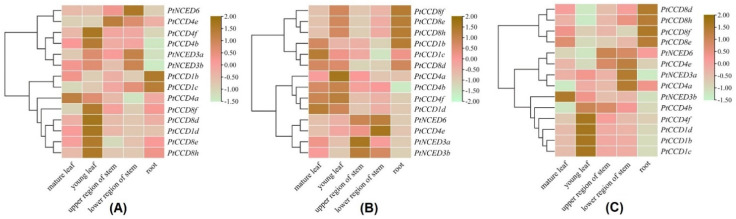
The qRT-PCR analysis of *PtCCDs* expression patterns in different tissues of poplar varieties including *P. trichocarpa* (**A**), ‘Nanlin 895’ (**B**), and ‘Shanxinyang’ (**C**). The gene expression levels in mature and young leaves, upper and lower regions of stems and roots were identified using qRT-PCR. Three independent experiments were performed. The data are normalized to poplar *Ptactin* (XM-006370951). *PtCCD* transcript levels were normalized to that in mature leaves; color scale, log2-fold change.

To gain insight into expression profiling of *PtCCDs* in different tissues, the qRT-PCR was used to illustrate gene expression levels in mature and young leaves, the upper and lower region of stems, and roots. There was a dominant gene expression divergence over different tissues and distinct expression patterns within poplar varieties (Figure 7). For tissue-specific expression patterns of *PtCCDs* in *P. trichocarpa*, the *PtCCD1b* and *1c* were highly expressed in roots. The relatively higher expression accumulations of *PtNCED3a*, *3b,* and *6* were presented in a lower region of stems. The mRNA transcript levels of *PtCCD4e* were abundant in the upper region of stems. The higher expression levels of *PtCCD1d*, *4b*, *4f*, *8d*, *8e*, *8f*, and *8h* were shown in young leaves (Figure 7A). In addition, for expression profiling of *PtCCDs* in ‘Nanlin 895’ (*P. deltoides* × *P. euramericana*), *PtCCD1b*, *8f*, *8e*, and *8h* showed higher abundances in roots, the higher expression levels of *PtNCED6* and *PtCCD4e* were accumulated in the lower region of stems, *PtCCD4a*, *4b,* and *4f* showed higher mRNA expression in young leaves, and *PtCCD1c* and *1d* showed higher expression levels in the mature leaves (Figure 7B). In addition, for expression patterns of *PtCCDs* in ‘Shanxinyang’ (*P. davidiana* × *P. bolleana* Loucne), 4 (*PtCCD8d*, *8e*, *8f*, and *8h*) were illustrated to be highly expressed in roots, 6 (*PtCCD1b*, *1c*, *1d*, *4b*, and *4f*) shared the highest expression in young leaves, and *PtNCED3a* and *PtCCD4a* and *4e* were highly expressed in the lower region of stems (Figure 7C).

To explore the putative relationship among the *PtCCD* genes, the clustering analysis was performed based on the *PtCCD* expression patterns. It is distinct that *PtCCD* genes in *P. trichocarpa*, ‘Nanlin 895’, and ‘Shanxinyang’ can be clustered into different clades, respectively. For example, the *PtCCD8d*, *8e*, *8f*, *8h*, and *1d* clustered into the same clade possessed relatively lower expression levels in young leaves, while the *PtCCD1b* and *1c* clustered into the same clade showed the higher accumulations in roots. In addition, clustering analysis in ‘Nanlin 895’ indicated that *PtCCD8e*, *8f*, and *8h* clustered into the same clade shared similar tissue-specific expression patterns. In addition, *PtCCD* genes transcript patterns in ‘Shanxinyang’ were divided into three clusters. Cluster 1, 2, and 3 displayed higher expression levels in roots, stems, and leaves, respectively. All above observations revealed that *PtCCD* genes showed tissue-specific expression patterns in poplar, and diverse expression patterns of *PtCCDs* are presented at poplar varieties. The results could illustrate that *PtCCD* genes may be involved in various physiological processes, and the function of *PtCCD* genes possibly experience evolution in response to different environments.

### 2.8. *PtCCD* Expression in Response to Abiotic Stress

**Figure 8:**
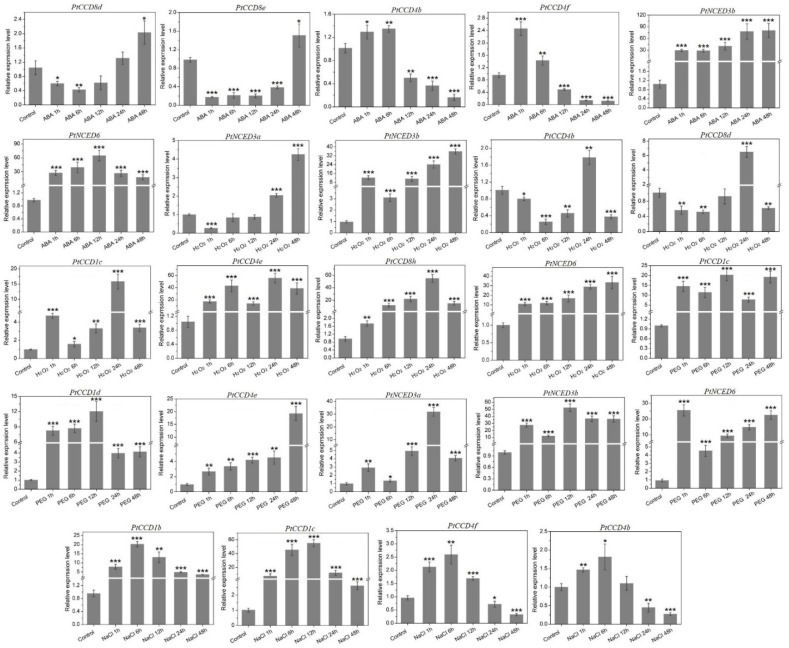
The qRT-PCR analysis of the *PtCCD* expression levels induced by abiotic treatments including ABA, H_2_O_2_, PEG_6000_, and NaCl. The vertical bars are representative of SD. Asterisk represents significant difference (*t*-test, * *p* < 0.05, ** *p* < 0.01, and *** *p* < 0.001). Three independent experiments were performed. The data are normalized to poplar *Ptactin* (XM-006370951). *PtCCD* transcript levels were normalized to that in untreated leaves (control).

To illustrate the expression profiling of *PtCCD* genes under abiotic stress, time-course changes were analyzed according to the qRT-PCR. Figure 8 and Supplemental Figure S8 shows *PtCCD* gene expression levels were distinctly affected by abiotic stress, and there are connections and differences in the expression pattern of each *PtCCD* member. Under the ABA treatment, the expression of *PtCCD8d* and *8e* were decreased during the early ABA treatment and recovered at 12 and 24 h, respectively; *PtCCD4b* and *4f* expression was up-regulated with 6h and maintained low expression level during 12–48 h; and other *PtCCD* genes showed relatively higher expression levels within the ABA treatment period, especially *PtNCED3b* and *6*. Under the H_2_O_2_ treatment, the expression abundances of *PtNCED3a* and *3b* and *PtCCD4b* and *8d* were downregulated during 0–12 h, and the higher expression level was found at 24 h; *PtCCD1c*, *4e*, *8h,* and *PtNCED6* were upregulated and displayed the higher abundances after H_2_O_2_ stress, but difference existed in reaching the highest expression level. Under the PEG_6000_ treatment, the expression levels of *PtCCD4b* and *4f* were increased firstly and then kept lower expression levels, while the transcript levels of *PtCCD8d*, *8e*, and *8f* displayed the negative results. Expression accumulations of *PtCCD1c*, *1d*, and *4e* and *PtNCED3a*, *3b,* and *6* were dominantly induced during the period of PEG_6000_ treatment. Under the NaCl treatment, the higher accumulations of *PtCCD1b* and *1c* were illustrated during 0–48 h; the expression levels of *PtCCD4f* and *4b* were significantly promoted in early-stage and were dominantly decreased in the later stage. In addition, it is distinct noting that some *PtCCD* genes clustered into the same clade had relatively similar expression profiling. For example, *PtNCED3a* and *3b* had similar mRNA transcript profiling under the H_2_O_2_ treatment. In addition, the expression levels of *PtCCD8d*, *8e*, and *8f* were generally downregulated in the early stage of PEG_6000_ treatment and then increased (Supplemental Figure S8). The above results showed that poplar *CCD* genes might be involved in response to abiotic and hormone stresses, and different *PtCCD* members had divergent response patterns.

## 3. Discussion

The CCD family containing the RPE65 domain participates in the carotenoid metabolic pathway. Almost all eukaryotes contain the CCD family, especially various *CCD* genes in plants, from yeast to human beings. For example, 9, 11, 9, and 19 *CCD* genes were identified in Arabidopsis [17], rice [35], tomato [51], and grape [36], respectively. In this study, 23 *PtCCD* genes were identified from poplar genome, and the compositions of PtCCD1, PtCCD4, PtCCD8, and PtNCED classes differed with other species. For example, each Arabidopsis CCD class contained one *CCD* gene. Rice contained above fourth CCD classes, and CCD8 class contained 8 *OsCCD* genes. While the PtCCD1, 4, and 8 classes contained 4, 6, and 8 *PtCCD* genes, respectively. Compared with Arabidopsis CCD classes, the number of poplar *CCD* genes was significantly increased, suggesting that poplar *CCD* genes might experience gene expansion in the process of evolution. In addition, Arabidopsis NCED subfamily contained *AtNCED2*, *3*, *5*, *6*, and *9*, while the poplar NCED subfamily only contained *NCED3* and *6*. Although the number of *PtNCED* genes was lower than the number of *AtNCED* genes, the NCED subfamily was closely associated with the CCD1 class, and *PtCCD1* genes were dominantly more than *AtCCD1* genes. It has been speculated that parts of PtCCD1 members may make up for the lack of PtNCED function. PtCCD family members are unevenly distributed on 10 poplar chromosomes, of which chromosome 18 contained the relatively more *PtCCD* genes, and only one *PtCCD* gene was identified to be distributed on chromosomes 03, 04, and 05. Moreover, 5 gene pairs of 23 *PtCCD* genes had a homologous evolutionary relationship, including tandem and segmental replication. Among them, *PtCCD4d* and *PtCCD4e*, and *PtCCD8e* and *PtCCD8f* originated from gene tandem replication events, while *PtCCD1a* and *PtCCD1d*, *PtCCD4a*, and *PtCCD4c*, and *PtNCED3a* and *PtNCED3b* originated from segmental replication events, which suggested that gene replication events may result in expansion of *PtCCD* gene family. The Ka/Ks ratios of 5 *PtCCD* pairs were far lower than 1, indicating that they have undergone strong purification selection in the process of evolution. In addition, the harmful non-synonymous substitution disappeared in the evolutionary process, indicating that gene duplication is the main reason for *PtCCD* gene expansion.

In Arabidopsis, AtCCD7 and AtCCD8 could catalyze β-carotene to form caprolactone, the precursor of strigolactone (SL) reported being involved in plant growth and development [52]. In addition, the CsCCD7 and CsCCD8 in saffron were identified to affect the synthesis of SL and control bud sprouting [53]. Similarly, CCD7 and CCD8 in kiwifruit [54], tomato [55], and rice [56] were involved in the regulation of cell senescence, root growth, branch, tiller, and flower organ morphogenesis. In this study, PtCCD7 and PtCCD8 groups showed a closer evolutionary relationship, and gene structures and motif compositions were usually similar in the same clade. Due to the conservation of *CCD* genes, *CCD* genes with similar functions are often clustered in the same clade, which provided an essential basis for studying the role of PtCCD7 and PtCCD8 classes. NCEDs were rate-limiting enzymes of ABA biosynthesis, and ABA as a crucial signal molecule participated in plant growth and development and stress response [57,58]. NCED could be associated with the contents of endogenous ABA and play an essential role in stress tolerance. In Arabidopsis, *AtNCED3* expression was induced by drought stress and participated in response to drought treatment by regulating leaf transpiration rate and controlling the level of endogenous ABA [22]. Also, AtNCED6 and AtNCED9 were involved in ABA biosynthesis during seed development [59]. CsNCED from *Crocus sativus* was closely associated with the content of endogenous ABA under salt, low temperature, and drought stresses [28]. In the present study, PtNCED2, 3, and 6 had similar motif compositions and gene structures with AtNCED2, 3, and 6, respectively, which suggested that PtNCED2, 3, and 6 may be involved in accumulations of endogenous ABA and response to abiotic stress. Compared with the PtCCD subfamily, a part of PtNCED clades was absent from the PtNCED subfamily, which indicated that functions of some PtNCEDs are replaced in the process of poplar evolution. The cleavage of carotenoids catalyzed by CCD4 was related to the coloring of pulp and flower organs. Overexpression of Arabidopsis *AtCCD4* in rice decreased contents of β-carotene and lutein and improved β-violone accumulation [60]. The loss function of *CCD4* led to changes in the color of fruit and flower organs. For example, loss-of-function of *CCD4* resulted in the change of azalea petal color from yellow to white [61] and the change of *Eustoma grandiflorum* petal color from light yellow to white [62]. The previous studies showed differences in the cleavage sites of carotenoids and substrates catalyzed by CCD4. In general, CCD4 cleaved carotenoids at 9′–10′ double bonds, while the cleavage position of CcCCD4b1 in citrus was 7′–8′ double bond, and the product was β-citraurin, β-citaurinene as the unique C_30_ carotenoids [63]. VvCCD4a from grape could catalyze red lycopene to form 6-methyl-5-heptene-2-one. In this study, 6 *PtCCD4s* were identified from poplar genome, and PtCCD4b and 4f had the closer relationship with AtCCD4. All the observations indicated that PtCCD4b and 4f might be involved in poplar pigment formation, and the activity of other PtCCD4 members may be complex. It should be comprehensively analyzed in further studies.

To identify the putative functions of PtCCDs associated with poplar growth and development, the transcript profiling of *PtCCDs* in young and mature leaves, upper and lower region of stems, and roots of poplar varieties were analyzed using qRT-PCR. The divergences in the *PtCCD* expression patterns were observed in poplar varieties, and even the same *PtCCD* was identified to have different expression levels in different poplar varieties. Those results indicated that the same PtCCD might be involved in various physiological processes in diverse poplar varieties. The Arabidopsis and petunia *CCD1* genes were highly expressed in all tested tissues [17,64], while *PtCCD1* genes had high expression levels in leaves of poplar varieties. *PtCCD1* expression patterns were inconsistent with its homologs Arabidopsis and petunia *CCD1* expression patterns, implying that *PtCCD1* genes participate in different physiological processes from Arabidopsis and petunia *CCD1* genes. Although the PtCCD1 class and PtNCED subfamily had the closer evolutionary relationship, the higher expression levels of *PtNCED3a* and *PtNCED6* were found in stems, and *PtCCD1* members highly expressed in leaves of poplar varieties, suggesting that PtNCED family and PtCCD1 class may have divergent functions in stems and leaves. In addition, *PtCCD7* gene was orthologous with *AtCCD7* gene, and previous studies showed *CCD7* has a distinct expression accumulation in roots [65,66]. Interestingly, in this study, the relatively lower expression levels of *PtCCD7* were identified in all tested tissues, which suggested that PtCCD7 in poplar may have different functions with *A. thaliana* CCD7 regarding root development and metabolite synthesis. However, the precise role and related regulation mechanism of PtCCD7 need to be confirmed in future studies. In addition, *PtCCD8* members had been confirmed to have the higher expression levels in *P. trichocarpa* young leaves, while they were identified to have the expression abundances in ‘Shanxinyang’ and ‘Nanlin 895’ roots. These results suggested that PtCCD8 members have divergent functions in the leaves and roots development of poplar varieties.

Since little knowledge focuses on the functions of the poplar CCD genes in regulating abiotic stress responses, the *PtCCDs* expression patterns in response to abiotic stress were illustrated. Previous studies have pointed out that the CCDs play an essential role in ABA synthesis and ABA signal transduction in response to diverse pressures [34,40]. This study identified various kinds of cis-elements involved in hormone-responsive and stress-related elements in the *PtCCD* promoters. *PtCCDs* mRNA levels in leaves induced by abiotic stress were investigated using qRT-PCR. *PtNCED3* and *6* expression abundances improved under the ABA treatment, similar to *AtNCED* gene accumulations caused by ABA treatment [67]. Although the distinct divergence was identified in the tissue-special expression of *PtCCD1* class, the *PtCCD1* members expression levels were also significantly up-regulated by ABA treatment. Compared with the previous conclusion that soybean *CCD7* and *CCD8* gene dominantly respond to ABA treatment [39], the *PtCCD8d* and *8e* expression levels were dominantly down-regulated at the early stage of ABA treatment, and *PtCCD8f* and *8h* expression levels were accumulated at the early stage of ABA treatment. Accordingly, *PtCCD1* members expression was significantly improved after ABA treatment, and even *PtCCD1a* expression abundance underwent a 56-fold increase after a 48 h ABA treatment. These results suggested that PtCCDs may play an essential role in response to ABA treatment. The precise regulation mechanism of plant stress responses by the ABA signal complex network might need to be explored in further study. Osmotic stress could change the plant physiological processes and affect plant growth and development by decreasing the photosynthetic rate and transpiration [68]. Also, under osmotic stress, the level of membrane lipid peroxidation was increased significantly, which destroyed the plant cell membrane and affected cell integrity [69,70]. Generally, osmotic stress significantly increased the contents of superoxide anion and endogenous H_2_O_2_ in the plant. Excessive accumulation of superoxide anion would cause irreversible damage to the cell membrane and seriously inhibit the progress of photosynthesis [71]. To explore the putative physiological changes caused by osmotic stress, the expression patterns of *PtCCDs* under the NaCl, PEG_6000_, and H_2_O_2_ were analyzed. The expression levels of *PtNCEDs* were considerably upregulated under osmotic stress, and differences only existed in expression trends with time-course. Those results were consistent with the higher expression of *Brassica rapa NCED* under the osmotic stress [40].

In addition, apple *MdCCD8a* expression levels were significantly improved under salt and drought treatments, and the soybean *CCD8* expression was decreased during drought and salt stresses. *PtCCD8s* showed diverse transcript levels under the NaCl and PEG_6000_ treatments. For example, *PtCCD8f* expression experienced an 11-fold increase in response to a 48-h PEG_6000_ treatment, while the peak of *PtCCD8f* expression was identified in 12 h under the H_2_O_2_ treatment. In addition, under the PEG_6000_ treatment, *PtCCD4b* and *4f* expression levels were up-regulated from 1 to 6 h, but there was distinct down-regulation of their expression levels from 12 to 48 h. However, under the H_2_O_2_ treatment, *PtCCD4b* expression level was significantly higher than in control, except its expression at 24 h. It was noteworthy that PtCCDs have different response abilities under ABA treatment and osmotic stress, indicating that PtCCDs play vital roles in abiotic stress. This study lays the foundation for identifying the biological functions of PtCCDs and helps to find stress-resistant gene resources.

## 4. Materials and Methods

### 4.1. Identification and Classification of Poplar *CCD* Genes

The RPE65 (PF03055) was achieved from the Pfam database (http://pfam.xfam.org/ (accessed on 28 July 2021)). The sequence information and innovation of poplar, Arabidopsis, rice, and willow were downloaded from the Phytozome database (https://phytozome-next.jgi.doe.gov/ (accessed on 28 July 2021)). The RPE65 is considered as a query to search the putative CCD members from the poplar genome. In addition, the Arabidopsis AtCCDs as a query to search the PtCCD members. Then, the SMART database and NCBI Conserved Domain Search online were applied further to verify the conserved domains in putative PtCCD members. Based on the homologous relationship with AtCCDs, the PtCCD members were named CCD1s, 4s, 7, 8s, and NCEDs.

### 4.2. Evolutionary Relationship and CCD Sequence Analysis

To illustrate the characterizations and putative functions of PtCCDs, multiple sequence alignment was performed using ClustalX2 software. In addition, according to the neighbor-joining (NJ) method, a phylogenetic tree on PtCCDs, AtCCDs, and OsCCDs evolutionary relationship was constructed. In addition, the poplar, Arabidopsis, rice annotation, and whole-genome information were applied to build the *CCD* gene structures. The MEME was used to identify the CCD motif compositions and distributions. Finally, all those generated files related to *CCD* gene structures and motifs were visualized using TBtools software [72,73].

### 4.3. Chromosomal Localization and Collinearity Analysis of *PtCCDs*

According to the *PtCCDs* annotation, the 23 *PtCCD* genes were mapped onto poplar chromosomes. The TBtools with MCScanX was applied to analyze tandem and segmental duplication events of *PtCCDs*, and TBtools with synteny visualization was used to visualize the collinearity relationship. In addition, the TBtools with a simple Ka/Ks calculator was applied to calculate Ka/Ks values between gene pairs. In addition, MCScanX was also used to identify the gene pairs with collinearity relationship among poplar, Arabidopsis, rice, and willow. Similarly, TBtools was applied to visualize syntenic blocks of orthologous genes.

### 4.4. Analyses of 3D Structures and Cis-Elements

The SWISS-MODEL (https://swissmodel.expasy.org/ (accessed on 28 July 2021)) was used to predict the structures of PtCCDs, and the α-helix, random coil, and strand PtCCDs were represented using Chimera software. The 2000 bp sequences upstream of the translation start sites of *CCD* genes were obtained from poplar, Arabidopsis, and rice genome database, and Plant CARE online tool was applied to predict cis-elements of *CCD* genes.

### 4.5. Plant Treatments and qRT-PCR Analysis

The leaves, stems, and roots harvested from the *P. trichocarpa*, ‘Shanxinyang’ (*P. davidiana* × *P. bolleana* Loucne), and ‘Nanlin 895’ (*P. deltoides* × *P. euramericana*) were used for tissue-specific gene expression analysis. Additionally, the leaves of ‘Nanlin 895’ grown on MS medium were treated with 2 mM of H_2_O_2_, 10% PEG_6000_, 200 mM of NaCl, and 200 μM of ABA, and leaves were collected at 0 (untreated leaves served as a control), 1, 6, 12, 24, and 48 h after each treatment. The abiotic stress treatments were performed with three replicates, with three poplars per replicate. Leaves harvested from treated and untreated poplars were stored at −80 °C.

Total RNA was extracted from various tissues and treated and untreated leaves using an RNA extraction kit (Takara, Japan). The reverse transcriptase (Takara, Japan) was used to synthesize first-strand cDNA of poplar. The UltraSYBR Green I Mixture (CWBIO, China) with 10 μL of Green I Mixture in a 20-μL reaction volume was applied to identify the *PtCCD* gene expression patterns. The Primer3.0 online tool was used to design the primers used in qRT–PCR. The qRT–PCR procedure was as follows: 95 °C for 10 min; 40 cycles of 94 °C for 10 s, 60 °C for 30 s, and 72 °C for 30 s. The relative *PtCCD* expression levels were identified according to the 2^−ΔΔCT^ method, with the *Ptactin* (XM-006370951), considered the internal control.

## 5. Conclusions

In this study, the characterizations and putative functions of 23 poplar CCD members were identified. These genes were divided into PtCCD1, PtCCD4, PtCCD7, PtCCD8, and PtNCED classes based on the molecular phylogenetic tree. The *PtCCD* gene structures and conserved motifs were also embodied in the evolutionary relationship. The 5 *PtCCD* pairs of homologous genes were identified in poplar genome, and 5 or 16 *CCD* pairs of orthologous genes were illustrated between poplar and Arabidopsis or willow, respectively. In addition, various kinds of stress-responsive cis-elements were identified in the promoters of *PtCCDs*, indicating that PtCCDs have a hand in comprehensive stress resistances. In addition, *PtCCDs* exhibited tissue-special expression patterns in poplar, implying they might be involved in divergent tissue and organ developments. Many *PtCCDs* expression levels were affected by ABA treatment and osmotic stress, suggesting that they may participate in ABA signal transduction or play an essential role in response to osmotic stress. The putative regulation mechanism of PtCCDs in response to abiotic and biotic stresses in poplar was predicted.

On one hand, abiotic and biotic stresses induce higher abundances of PtCCDs in poplar. *PtNCEDs* encode key enzymes for biosynthesis of ABA, ultimately causing the producing ABA signal transduction, resulting in abiotic and biotic stress resistance. On the other hand, other CCDs can catalyze carotenoids to form apocarotenoid substrates in response to stresses (Supplemental Figure S8). These will be beneficial for exploring PtCCD functions and potential regulatory mechanisms and lay the basis for illustrating corresponding gene networks involved in osmotic stress.

## Figures and Tables

**Table 1:** The poplar, Arabidopsis, and rice *CCD* accession numbers and gene names.

*Arabidopsis thaliana*		*Oryza sativa*		*Populus trichocarpa*	
Accessions	Gene name	Accessions	Gene name	Accessions	Gene name
AT3G63520	*AtCCD1*	LOC_Os12g44310	*OsCCD1*	Potri.001G265400	*PtCCD1a*
				Potri.001G265600	*PtCCD1b*
				Potri.001G265900	*PtCCD1c*
				Potri.009G060500	*PtCCD1d*
AT4G19170	*AtCCD4*	LOC_Os02g47510	*OsCCD4a*	Potri.004G190700	*PtCCD4a*
				Potri.005G069100	*PtCCD4b*
				Potri.009G151900	*PtCCD4c*
		LOC_Os12g24800	*OsCCD4b*	Potri.009G152200	*PtCCD4d*
				Potri.009G152300	*PtCCD4e*
				Potri.019G093400	*PtCCD4f*
AT2G44990	*AtCCD7*	LOC_Os04g46470	*OsCCD7*	Potri.014G056800	*PtCCD7*
AT4G32810	*AtCCD8*	LOC_Os01g54270	*OsCCD8a*	Potri.006G239200	*PtCCD8a*
				Potri.006G239400	*PtCCD8b*
		LOC_Os09g15240	*OsCCD8b*	Potri.018G042650	*PtCCD8c*
				Potri.018G042900	*PtCCD8d*
		LOC_Os01g38580	*OsCCD8c*	Potri.018G043000	*PtCCD8e*
				Potri.018G043100	*PtCCD8f*
		LOC_Os08g28240	*OsCCD8d*	Potri.018G043400	*PtCCD8g*
				Potri.018G043500	*PtCCD8h*
AT4G18350	*AtNCED2*	LOC_Os12g42280	*OsNCED2*	Potri.011G084100	*PtNCED2*
AT3G14440	*AtNCED3*	LOC_Os07g05940	*OsNCED3*	Potri.001G393800	*PtNCED3a*
				Potri.011G112400	*PtNCED3b*
AT1G30100	*AtNCED5*				
AT3G24220	*AtNCED6*			Potri.003G176300	*PtNCED6*
AT1G78390	*AtNCED9*	LOC_Os03g44380	*OsNCED9*		

## Data Availability

Not applicable.

## Supplementary Materials

The following are available online at https://www.mdpi.com/article/10.3390/ijms23031418/s1.

## Abbreviations

Carotenoid cleavage dioxygenase: CCD; 9-cis epoxycarotenoid dioxygenases: NCED; abscisic acid: ABA; multiple collinearity scan toolkit: MCScanX; anaerobic inducible element: ARE; stress response element: STRE; MYB drought inducible binding site: MBS; DREB/CBF transcription factor recognition site: DRE; low-temperature response elements: LTR; auxin response elements: TGA-element; salicylic acid: SA; methyl jasmonate: MeJA; ABA response element: ABRE; cytochrome P: CYP; phytoene synthase: PSY; abscisic acid insensitive: ABI; gibberellic oxidase: GAox; thaumatin-like protein: TCP; strigolactone: SL; neighbor-joining: NJ.

## References

[1] Chen K., Li G.J., Bressan R.A., Song C.P., Zhu J.K., Zhao Y. (2020). Abscisic acid dynamics, signaling, and functions in plants. J. Integr. Plant Biol..

[2] Rodriguez-Concepcion M., Avalos J., Bonet M.L., Boronat A., Gomez-Gomez L., Hornero-Mendez D., Limon M.C., Meléndez-Martínez A.J., Olmedilla-Alonso B., Palou A. (2018). A global perspective on carotenoids: Metabolism, biotechnology, and benefits for nutrition and health. Prog. Lipid Res..

[3] Woitsch S., Romer S. (2003). Expression of xanthophyll biosynthetic genes during light-dependent chloroplast differentiation. Plant Physiol..

[4] Bartley G.E., Scolnik P.A. (1995). Plant carotenoids: Pigments for photoprotection, visual attraction, and human health. Plant Cell..

[5] Bouvier F., Isner J.C., Dogbo O., Camara B. (2005). Oxidative tailoring of carotenoids: A prospect towards novel functions in plants. Trends Plant Sci..

[6] Ilahy R., Siddiqui M.W., Tlili I., Montefusco A., Piro G., Hdider C., Lenucci M.S. (2018). When color really matters: Horticultural performance and functional quality of high-lycopene tomatoes. Crit. Rev. Plant Sci..

[7] Wang C., Qiao A., Fang X., Sun L., Gao P., Davis A.R., Liu S., Luan F. (2019). Fine mapping of lycopene content and flesh color related gene and development of molecular marker-assisted selection for flesh color in watermelon (*Citrullus lanatus*). Front. Plant Sci..

[8] Trivellini A., Ferrante A., Vernieri P., Mensuali-Sodi A., Serra G. (2011). Effects of promoters and inhibitors of ethylene and aba on flower senescence of *Hibiscus rosa-sinensis* L.. J. Plant Growth Regul..

[9] Chernys J.T., Zeevaart J.A. (2000). Characterization of the 9-cis-epoxycarotenoid dioxygenase gene family and the regulation of abscisic acid biosynthesis in avocado. Plant Physiol..

[10] Shu K., Liu X.D., Xie Q., He Z.H. (2016). Two faces of one seed: Hormonal regulation of dormancy and germination. Mol. Plant.

[11] Seo M., Koshiba T. (2002). Complex regulation of ABA biosynthesis in plants. Trends Plant Sci..

[12] Kuromori T., Sugimoto E., Shinozaki K. (2011). Arabidopsis mutants of AtABCG22, an ABC transporter gene, increase water transpiration and drought susceptibility. Plant J..

[13] McAdam S.A., Brodribb T.J., Banks J.A., Hedrich R., Atallah N.M., Cai C., Geringer M.A., Lind C., Nichols D.S., Stachowski K. (2016). Abscisic acid controlled sex before transpiration in vascular plants. Proc. Natl. Acad. Sci. USA.

[14] Islam M.M., Ye W., Matsushima D., Munemasa S., Okuma E., Nakamura Y., Biswas S., Mano J.I., Murata Y. (2016). Reactive carbonyl species mediate ABA signaling in guard cells. Plant Cell Physiol..

[15] Li X., Zhao J., Sun Y., Li Y. (2020). *Arabidopsis thaliana* CRK41 negatively regulates salt tolerance via H_2_O_2_ and ABA cross-linked networks. Environ. Exp. Bot..

[16] Kloer D.P., Ruch S., Al-Babili S., Beyer P., Schulz G.E. (2005). The structure of a retinal-forming carotenoid oxygenase. Science.

[17] Auldridge M.E., Block A., Vogel J.T., Dabney-Smith C., Mila I., Bouzayen M., Magallanes-Lundback M., DellaPenna D., McCarty D.R., Klee H.J. (2006). Characterization of three members of the Arabidopsis carotenoid cleavage dioxygenase family demonstrates the divergent roles of this multifunctional enzyme family. Plant J..

[18] Tan B.C., Joseph L.M., Deng W.T., Liu L., Li Q.B., Cline K., McCarty D.R. (2003). Molecular characterization of the Arabidopsis 9-cis epoxycarotenoid dioxygenase gene family. Plant J..

[19] Wang P., Lu S., Zhang X., Hyden B., Qin L., Liu L., Bai Y., Han Y., Wen Z., Xu J. (2021). Double NCED isozymes control ABA biosynthesis for ripening and senescent regulation in peach fruits. Plant Sci..

[20] Tan B.C., Schwartz S.H., Zeevaart J.A., McCarty D.R. (1997). Genetic control of abscisic acid biosynthesis in maize. Proc. Natl. Acad. Sci. USA.

[21] Schwartz S.H., Tan B.C., Gage D.A., Zeevaart J.A., McCarty D.R. (1997). Specific oxidative cleavage of carotenoids by VP14 of maize. Science.

[22] Iuchi S., Kobayashi M., Taji T., Naramoto M., Seki M., Kato T., Tabata S., Kakubari Y., Yamaguchi-Shinozaki K., Shinozaki K. (2001). Regulation of drought tolerance by gene manipulation of 9-cis-epoxycarotenoid dioxygenase, a key enzyme in abscisic acid biosynthesis in Arabidopsis. Plant J..

[23] Fan J., Hill L., Crooks C., Doerner P., Lamb C. (2009). Abscisic acid has a key role in modulating diverse plant-pathogen interactions. Plant Physiol..

[24] Changan S.S., Ali K., Kumar V., Garg N.K., Tyagi A. (2018). Abscisic acid biosynthesis under water stress: Anomalous behavior of the 9-cis-epoxycarotenoid dioxygenase1 (NCED1) gene in rice. Biol. Plantarum.

[25] Song S., Dai X., Zhang W.H. (2012). A rice F-box gene, Os Fbx352, is involved in glucose-delayed seed germination in rice. J. Exp. Bot..

[26] Hwang S.G., Chen H.C., Huang W.Y., Chu Y.C., Shii C.T., Cheng W.H. (2010). Ectopic expression of rice *OsNCED3* in Arabidopsis increases ABA level and alters leaf morphology. Plant Sci..

[27] Huang Y., Jiao Y., Xie N., Guo Y., Zhang F., Xiang Z., Wang R., Wang F., Gao Q., Tian L. (2019). OsNCED5, a 9-cis-epoxycarotenoid dioxygenase gene, regulates salt and water stress tolerance and leaf senescence in rice. Plant Sci..

[28] Ahrazem O., Rubio-Moraga A., Trapero A., Gómez-Gómez L. (2012). Developmental and stress regulation of gene expression for a 9-cis-epoxycarotenoid dioxygenase, CstNCED, isolated from *Crocus sativus* stigmas. J. Exp. Bot..

[29] Huang F.C., Molnár P., Schwab W. (2009). Cloning and functional characterization of carotenoid cleavage dioxygenase 4 genes. J. Exp. Bot..

[30] Baldermann S., Kato M., Kurosawa M., Kurobayashi Y., Fujita A., Fleischmann P., Watanabe N. (2010). Functional characterization of a carotenoid cleavage dioxygenase 1 and its relation to the carotenoid accumulation and volatile emission during the floral development of *Osmanthus fragrans* Lour. J. Exp. Bot..

[31] Yahyaa M., Bar E., Dubey N.K., Meir A., Davidovich-Rikanati R., Hirschberg J., Aly R., Tholl D., Simon P.W., Tadmor Y. (2013). Formation of norisoprenoid flavor compounds in carrot (*Daucus carota* L.) roots: Characterization of a cyclic-specific carotenoid cleavage dioxygenase 1 gene. J. Agric. food Chem..

[32] Ilg A., Beyer P., Al-Babili S. (2009). Characterization of the rice carotenoid cleavage dioxygenase 1 reveals a novel route for geranial biosynthesis. FEBS J..

[33] Schwartz S.H., Qin X., Loewen M.C. (2004). The biochemical characterization of two carotenoid cleavage enzymes from Arabidopsis indicates that a carotenoid-derived compound inhibits lateral branching. J. Biol. Chem..

[34] Alder A., Jamil M., Marzorati M., Bruno M., Vermathen M., Bigler P., Ghisla S., Bouwmeester H., Beyer P., Al-Babili S. (2012). The path from β-carotene to carlactone, a strigolactone-like plant hormone. Science.

[35] Vallabhaneni R., Bradbury L.M., Wurtzel E.T. (2010). The carotenoid dioxygenase gene family in maize, sorghum, and rice. Arch. Biochem. Biophys..

[36] Lashbrooke J.G., Young P.R., Dockrall S.J., Vasanth K., Vivier M.A. (2013). Functional characterisation of three members of the *Vitis vinifera* L. carotenoid cleavage dioxygenase gene family. BMC Plant Biol..

[37] Ohmiya A., Kishimoto S., Aida R., Yoshioka S., Sumitomo K. (2006). Carotenoid cleavage dioxygenase (CmCCD4a) contributes to white color formation in chrysanthemum petals. Plant Physiol..

[38] Rodrigo M.J., Alquézar B., Alós E., Medina V., Carmona L., Bruno M., Al-Babili S., Zacarías L. (2013). A novel carotenoid cleavage activity involved in the biosynthesis of Citrus fruit-specific apocarotenoid pigments. J. Exp. Bot..

[39] Wang R.K., Wang C.E., Fei Y.Y., Gai J.Y., Zhao T.J. (2013). Genome-wide identification and transcription analysis of soybean carotenoid oxygenase genes during abiotic stress treatments. Mol. Biol. Rep..

[40] Kim Y., Hwang I., Jung H.J., Park J.I., Kang J.G., Nou I.S. (2016). Genome-wide classification and abiotic stress-responsive expression profiling of carotenoid oxygenase genes in *Brassica rapa* and *Brassica oleracea*. J. Plant Growth Regul..

[41] Chen H., Zuo X., Shao H., Fan S., Ma J., Zhang D., Zhao C., Yan X., Liu X., Han M. (2018). Genome-wide analysis of carotenoid cleavage oxygenase genes and their responses to various phytohormones and abiotic stresses in apple (*Malus domestica*). Plant Physiol. Bioch..

[42] Kumar M., Kherawat B.S., Dey P., Saha D., Singh A., Bhatia S.K., Ghodake G.S., Kadam A.A., Kim H.U., Chung S.M. (2021). Genome-Wide Identification and Characterization of PIN-FORMED (PIN) Gene Family Reveals Role in Developmental and Various Stress Conditions in *Triticum aestivum* L.. Int. J. Mol. Sci..

[43] Tong T., Fang Y.X., Zhang Z., Zheng J., Zhang X., Li J., Niu C., Xue D., Zhang X. (2021). Genome-wide identification and expression pattern analysis of the KCS gene family in barley. Plant Growth Regul..

[44] Baruah P.M., Krishnatreya D.B., Bordoloi K.S., Gill S.S., Agarwala N. (2021). Genome wide identification and characterization of abiotic stress responsive lncRNAs in *Capsicum annuum*. Plant Physiol. Biochem..

[45] Kesawat M.S., Kherawat B.S., Singh A., Dey P., Kabi M., Debnath D., Saha D., Khandual A., Rout S., Ali A. (2021). Genome-wide identification and characterization of the brassinazole-resistant (BZR) gene family and its expression in the various developmental stage and stress conditions in wheat (*Triticum aestivum* L.). Int. J. Mol. Sci..

[46] Jiang M., Chen H., Liu J., Du Q., Lu S., Liu C. (2021). Genome-wide identification and functional characterization of natural antisense transcripts in Salvia miltiorrhiza. Sci. Rep..

[47] Wang M., Chen B., Zhou W., Xie L., Wang L., Zhang Y., Zhang Q. (2021). Genome-wide identification and expression analysis of the AT-hook Motif Nuclear Localized gene family in soybean. BMC Genom..

[48] Zhou Q., Li Q., Li P., Zhang S., Liu C., Jin J., Cao P., Yang Y. (2019). Carotenoid cleavage dioxygenases: Identification, expression, and evolutionary analysis of this gene family in tobacco. Int. J. Mol. Sci..

[49] Tuskan G.A., Difazio S., Jansson S., Bohlmann J., Grigoriev I., Hellsten U., Putnam N., Ralph S., Rombauts S., Salamov A. (2006). The genome of black cottonwood, *Populus trichocarpa* (Torr. & Gray). Science.

[50] Zhang J., Yuan H., Li Y., Chen Y., Liu G., Ye M., Yu C., Lian B., Zhong F., Jiang Y. (2020). Genome sequencing and phylogenetic analysis of allotetraploid *Salix matsudana* Koidz. Hortic. Res..

[51] Wei Y., Wan H., Wu Z., Wang R., Ruan M., Ye Q., Li Z., Zhou G., Yao Z., Yang Y. (2016). A comprehensive analysis of carotenoid cleavage dioxygenases genes in *Solanum lycopersicum*. Plant Mol. Biol. Rep..

[52] Bruno M., Vermathen M., Alder A., Wüst F., Schaub P., van der Steen R., Beyer P., Ghisla S., Al-Babili S. (2017). Insights into the formation of carlactone from in-depth analysis of the CCD 8-catalyzed reactions. FEBS Lett..

[53] Rubio-Moraga A., Ahrazem O., Pérez-Clemente R.M., Gómez-Cadenas A., Yoneyama K., López-Ráez J.A., Molina R.V., Gómez-Gómez L. (2014). Apical dominance in saffron and the involvement of the branching enzymes CCD7 and CCD8 in the control of bud sprouting. BMC Plant Biol..

[54] Ledger S.E., Janssen B.J., Karunairetnam S., Wang T., Snowden K.C. (2010). Modified CAROTENOID CLEAVAGE DIOXYGENASE8 expression correlates with altered branching in kiwifruit (*Actinidia chinensis*). New Phytol..

[55] Vogel J.T., Walter M.H., Giavalisco P., Lytovchenko A., Kohlen W., Charnikhova T., Simkin A.J., Goulet C., Strack D., Bouwmeester H.J. (2010). SlCCD7 controls strigolactone biosynthesis, shoot branching and mycorrhiza-induced apocarotenoid formation in tomato. Plant J..

[56] Kulkarni K.P., Vishwakarma C., Sahoo S.P., Lima J.M., Nath M., Dokku P., Gacche R.N., Mohapatra T., Robin S., Sarla N. (2014). A substitution mutation in *OsCCD7* cosegregates with dwarf and increased tillering phenotype in rice. J. Genet..

[57] Pei X., Wang X., Fu G., Chen B., Nazir M.F., Pan Z., He S., Du X. (2021). Identification and functional analysis of 9-cis-epoxy carotenoid dioxygenase (NCED) homologs in *G. hirsutum*. Int. J. Biol. Macromol..

[58] Zhang J., Zhang P., Huo X., Gao Y., Chen Y., Song Z., Wang F., Zhang J. (2021). Comparative phenotypic and transcriptomic analysis reveals key responses of upland cotton to salinity stress during postgermination. Front. Plant Sci..

[59] Lefebvre V., North H., Frey A., Sotta B., Seo M., Okamoto M., Nambara E., Marion-Poll A. (2006). Functional analysis of Arabidopsis *NCED6* and *NCED9* genes indicates that ABA synthesized in the endosperm is involved in the induction of seed dormancy. Plant J..

[60] Song M.H., Lim S.H., Kim J.K., Jung E.S., John K.M., You M.K., Ahn S.N., Lee C.H., Ha S.H. (2016). In planta cleavage of carotenoids by Arabidopsis carotenoid cleavage dioxygenase 4 in transgenic rice plants. Plant Biotechnol. Rep..

[61] Ureshino K., Nakayama M., Miyajima I. (2016). Contribution made by the carotenoid cleavage dioxygenase 4 gene to yellow colour fade in azalea petals. Euphytica.

[62] Liu H., Kishimoto S., Yamamizo C., Fukuta N., Ohmiya A. (2013). Carotenoid accumulations and carotenogenic gene expressions in the petals of *Eustoma grandiflorum*. Plant Breed..

[63] Zheng X., Zhu K., Sun Q., Zhang W., Wang X., Cao H., Tan M., Xie Z., Zeng Y., Ye J. (2019). Natural variation in CCD4 promoter underpins species-specific evolution of red coloration in citrus peel. Mol. Plant.

[64] Simkin A.J., Underwood B.A., Auldridge M., Loucas H.M., Shibuya K., Schmelz E., Clark D.G., Klee H.J. (2004). Circadian regulation of the PhCCD1 carotenoid cleavage dioxygenase controls emission of β-ionone, a fragrance volatile of petunia flowers. Plant Physiol..

[65] Booker J., Sieberer T., Wright W., Williamson L., Willett B., Stirnberg P., Turnbull C., Srinivasan M., Goddard P., Leyser O. (2005). MAX1 encodes a cytochrome P450 family member that acts downstream of MAX3/4 to produce a carotenoid-derived branch-inhibiting hormone. Dev. Cell.

[66] Delaux P.M., Xie X., Timme R.E., Puech-Pages V., Dunand C., Lecompte E., Delwiche C.F., Yoneyama K., Bécard G., Séjalon-Delmas N. (2012). Origin of strigolactones in the green lineage. New Phytol..

[67] Iuchi S., Kobayashi M., Yamaguchi-Shinozaki K., Shinozaki K. (2000). A stress-inducible gene for 9-cis-epoxycarotenoid dioxygenase involved in abscisic acid biosynthesis under water stress in drought-tolerant cowpea. Plant Physiol..

[68] Lawlor D.W., Cornic G. (2002). Photosynthetic carbon assimilation and associated metabolism in relation to water deficits in higher plants. Plant Cell Environ..

[69] Candan N., Tarhan L. (2012). Tolerance or sensitivity responses of *Mentha pulegium* to osmotic and waterlogging stress in terms of antioxidant defense systems and membrane lipid peroxidation. Environ. Exp. Bot..

[70] Zhang C., Shi S. (2018). Physiological and proteomic responses of contrasting alfalfa (*Medicago sativa* L.) varieties to PEG-induced osmotic stress. Front. Plant Sci..

[71] Li Q., Lv L.R., Teng Y.J., Si L.B., Ma T., Yang Y.L. (2018). Apoplastic hydrogen peroxide and superoxide anion exhibited different regulatory functions in salt-induced oxidative stress in wheat leaves. Biol. Plantarum.

[72] Chen C., Chen H., Zhang Y., Thomas H.R., Frank M.H., He Y., Xia R. (2020). TBtools: An integrative toolkit developed for interactive analyses of big biological data. Mol. Plant.

[73] Zhang J., zheng Shi S., Jiang Y., Zhong F., Liu G., Yu C., Lian B., Chen Y. (2021). Genome-wide investigation of the AP2/ERF superfamily and their expression under salt stress in Chinese willow (*Salix matsudana*). PeerJ.

