# Supplementary figures and images for: Comprehensive Analysis of Carotenoid Cleavage Dioxygenases Gene Family and Its Expression in Response to Abiotic Stress in Poplar
Source: Int J Mol Sci. 2022 Jan 26;23(3):1418. doi: 10.3390/ijms23031418 (PMC8836127; doi:10.3390/ijms23031418)

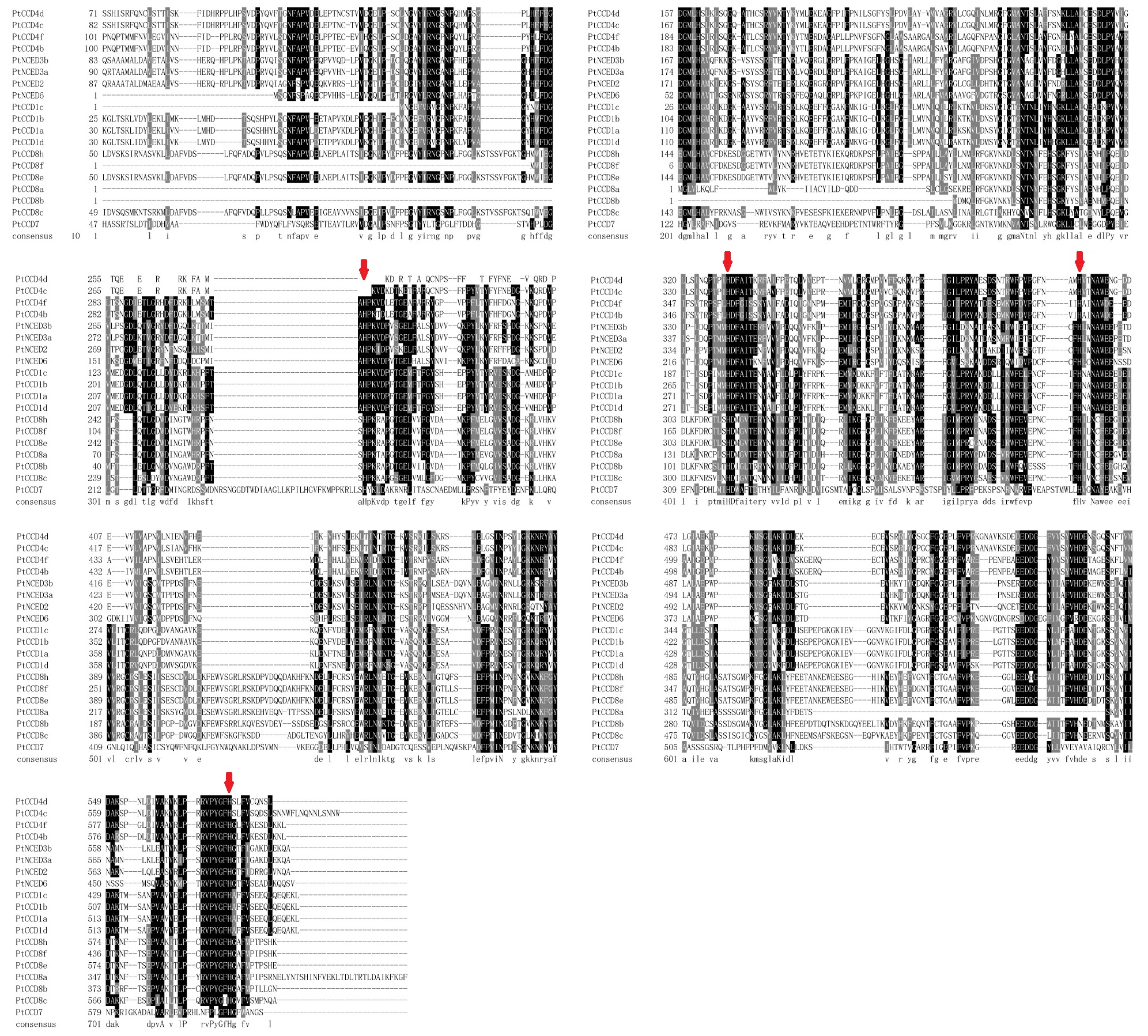

Supplement: Supplementary file 1 [file ijms-23-01418-s001.zip › supplemental figure s1.jpg]

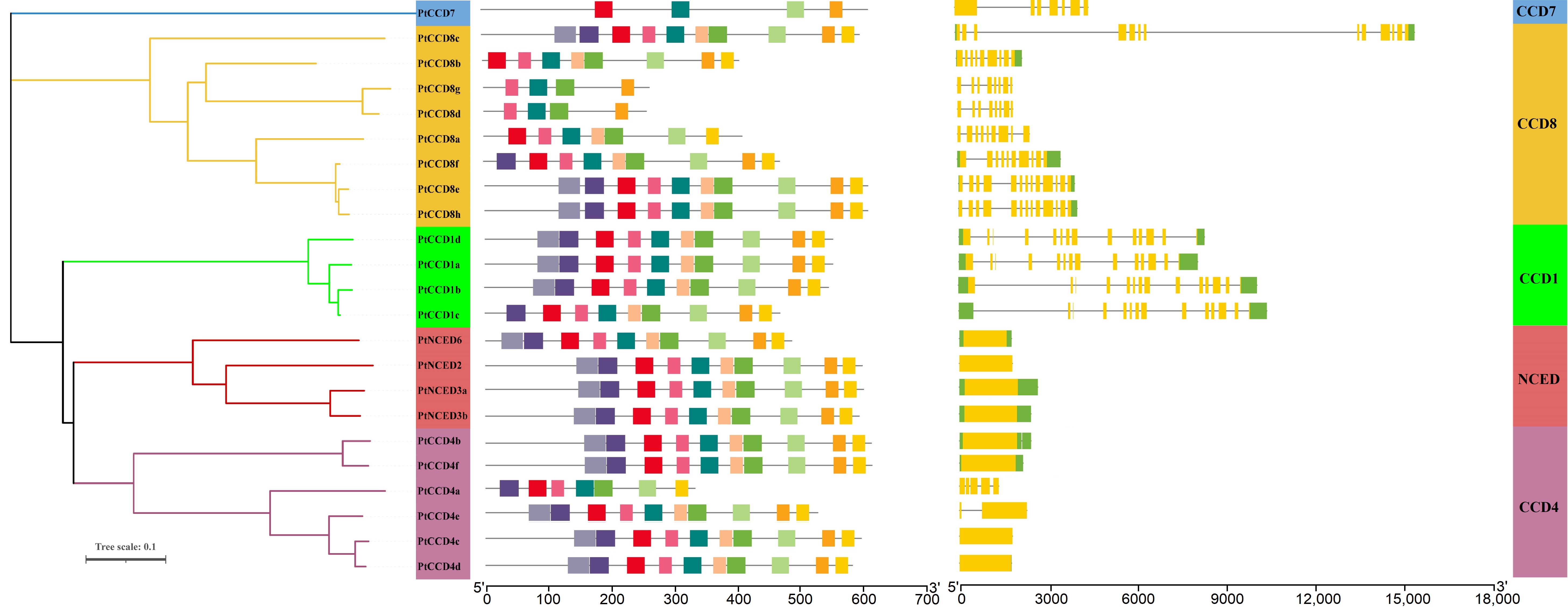

Supplement: Supplementary file 1 [file ijms-23-01418-s001.zip › supplemental figure s2.jpg]

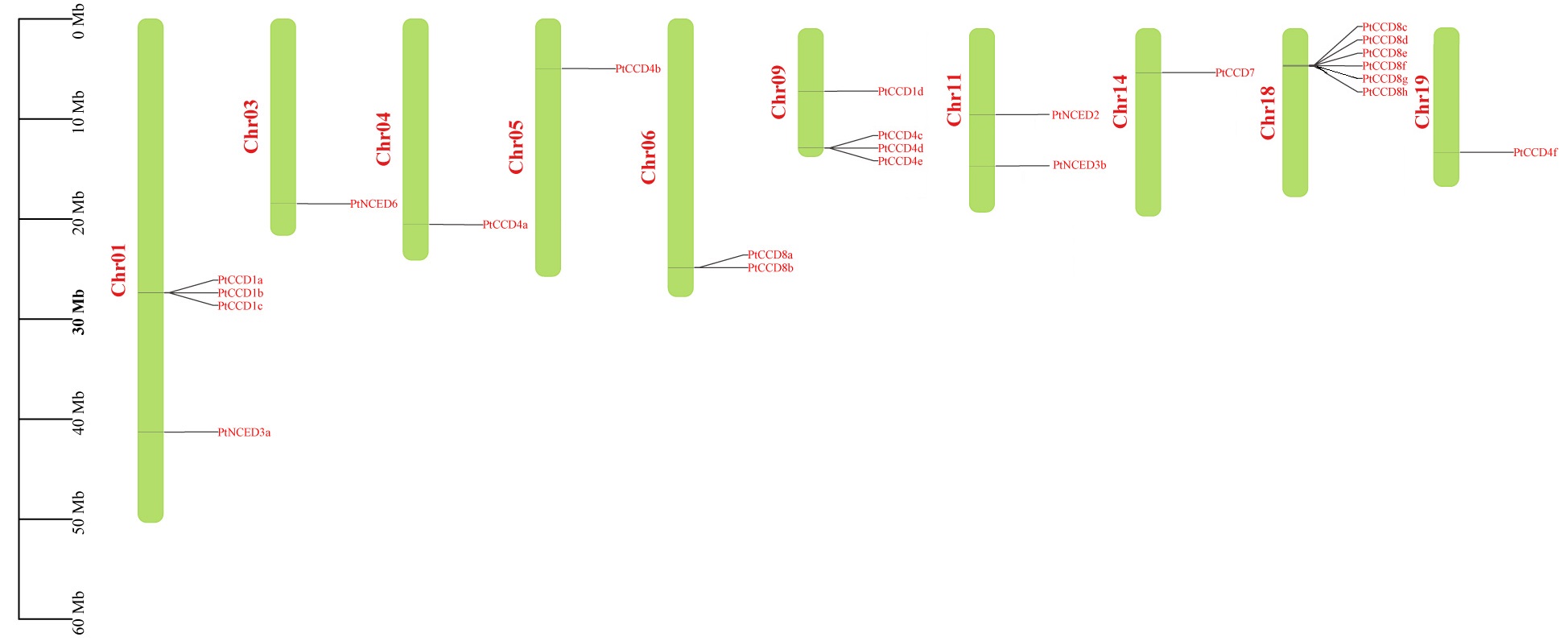

Supplement: Supplementary file 1 [file ijms-23-01418-s001.zip › supplemental figure s3.jpg]

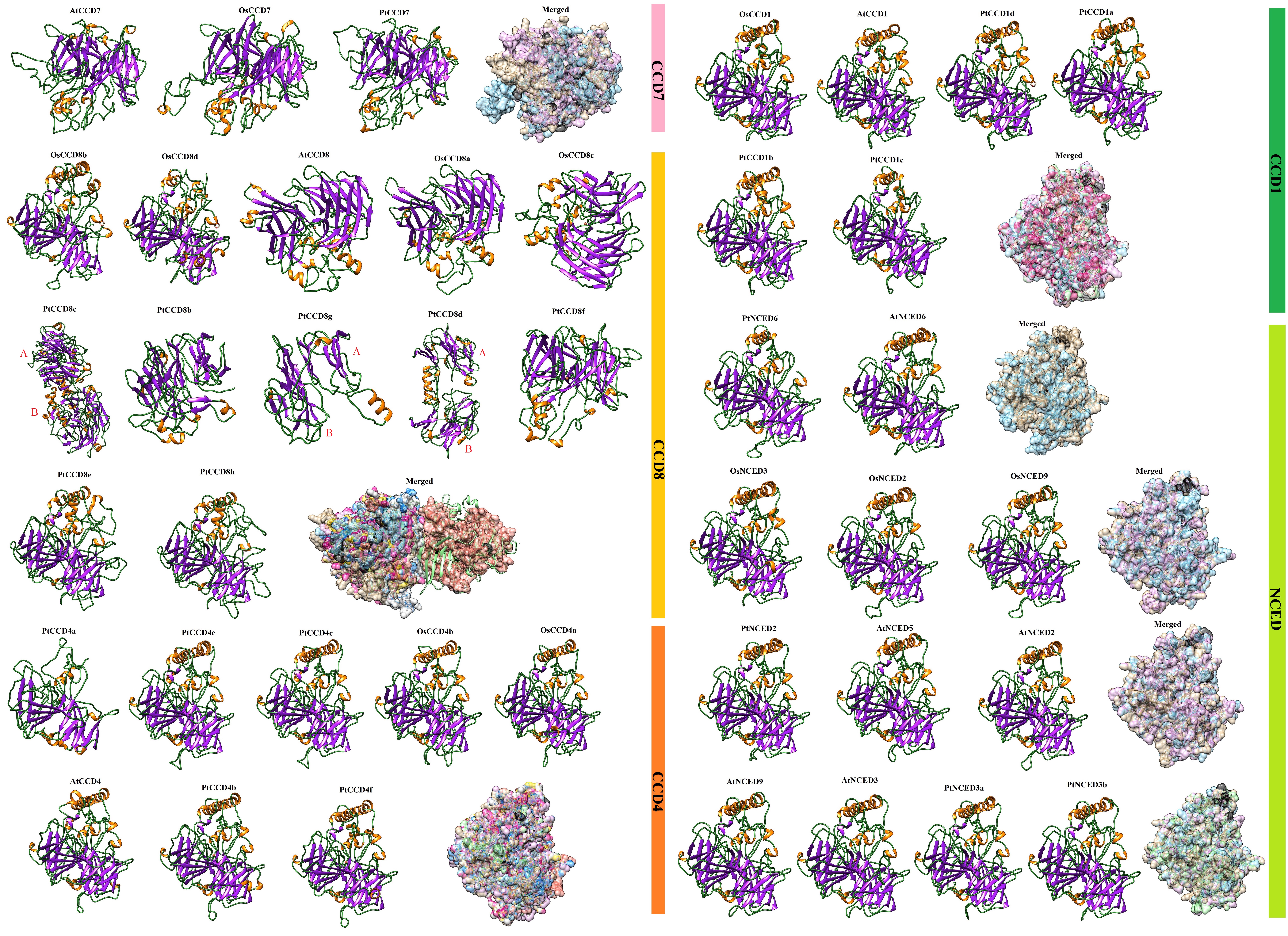

Supplement: Supplementary file 1 [file ijms-23-01418-s001.zip › supplemental figure s4.jpg]

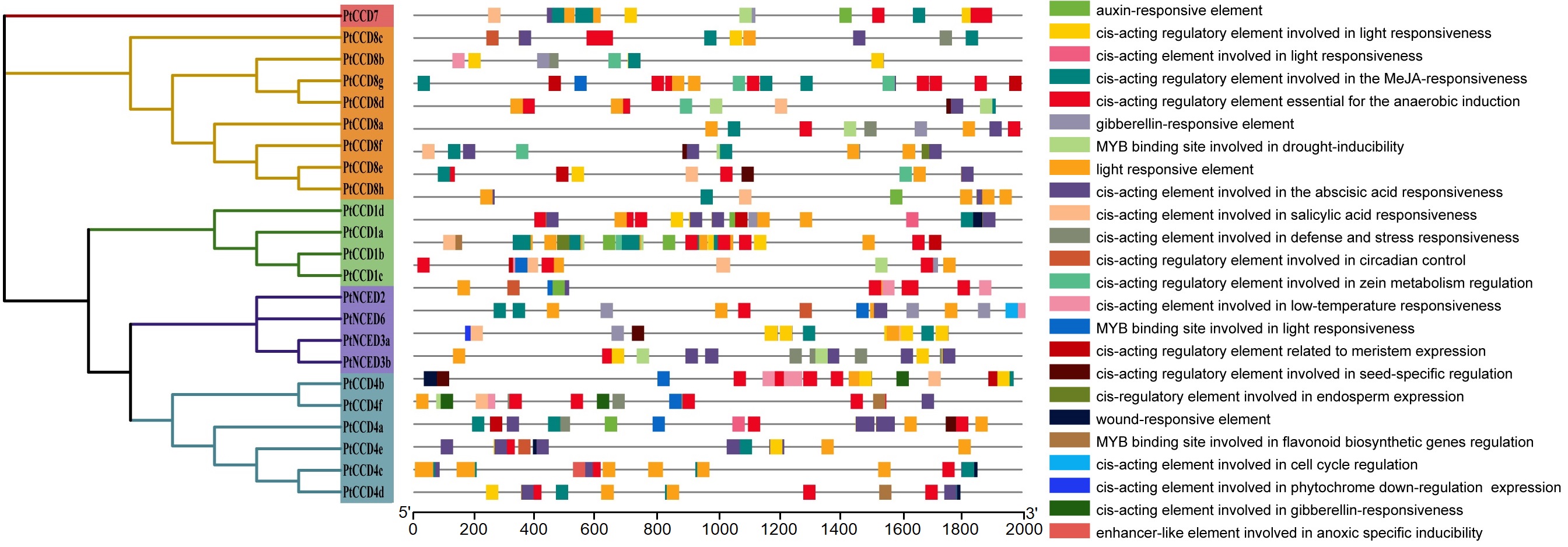

Supplement: Supplementary file 1 [file ijms-23-01418-s001.zip › supplemental figure s5.jpg]

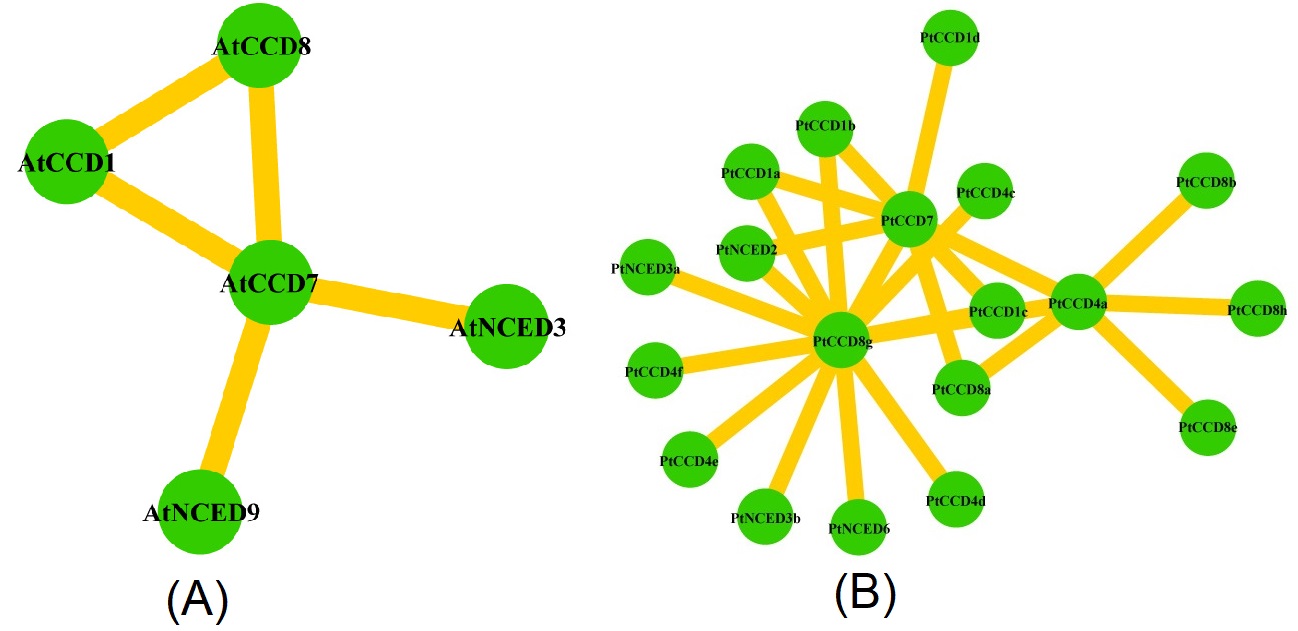

Supplement: Supplementary file 1 [file ijms-23-01418-s001.zip › Supplemental Figure s6.jpg]

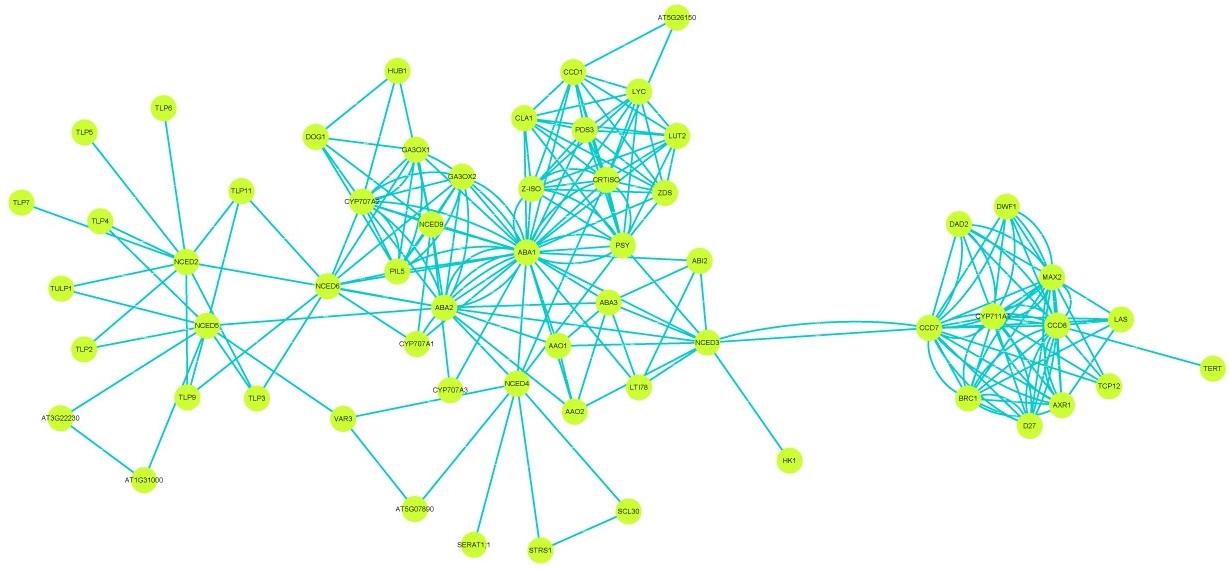

Supplement: Supplementary file 1 [file ijms-23-01418-s001.zip › Supplemental Figure s7.jpg]

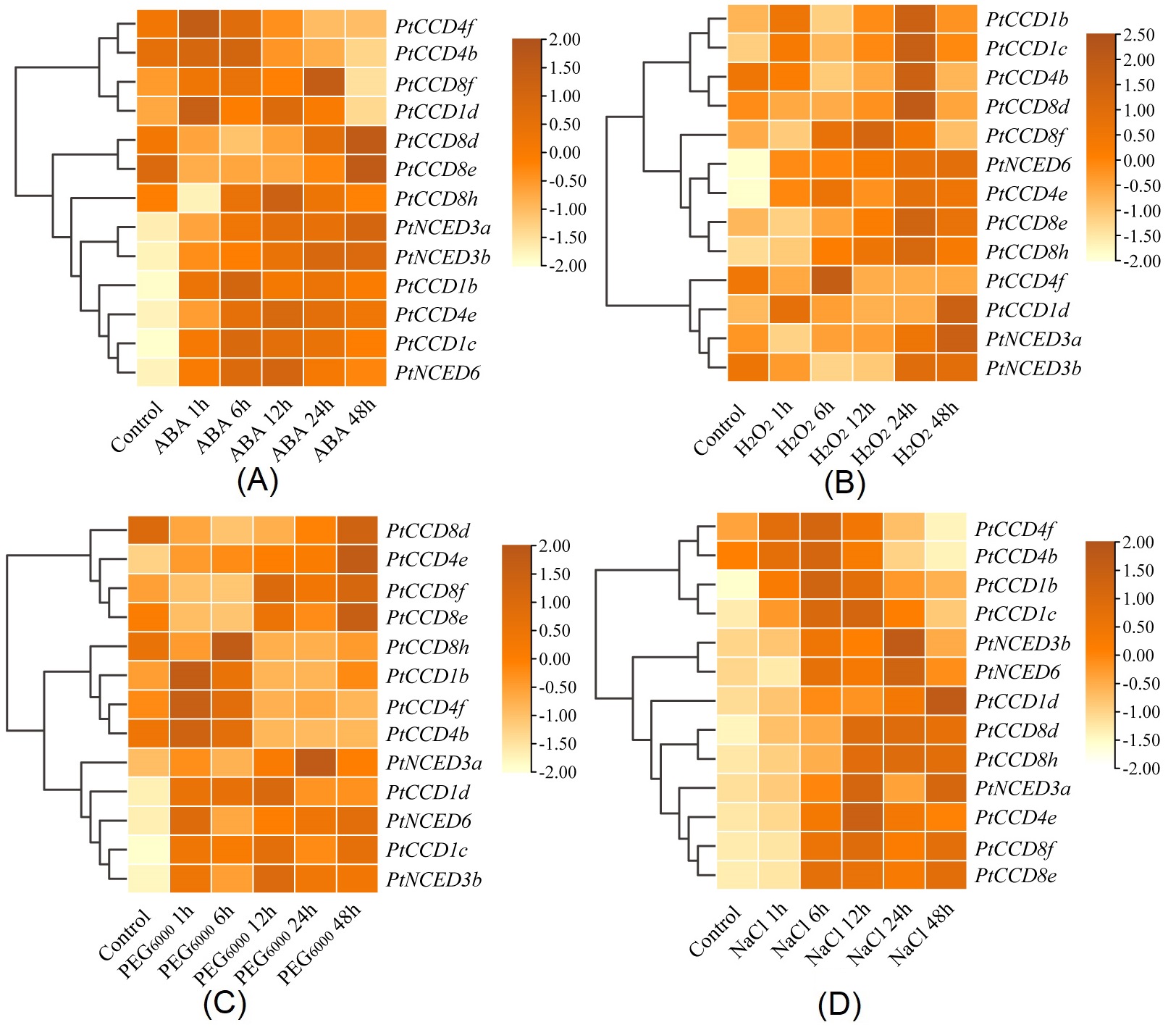

Supplement: Supplementary file 1 [file ijms-23-01418-s001.zip › Supplemental Figure s8.jpg]

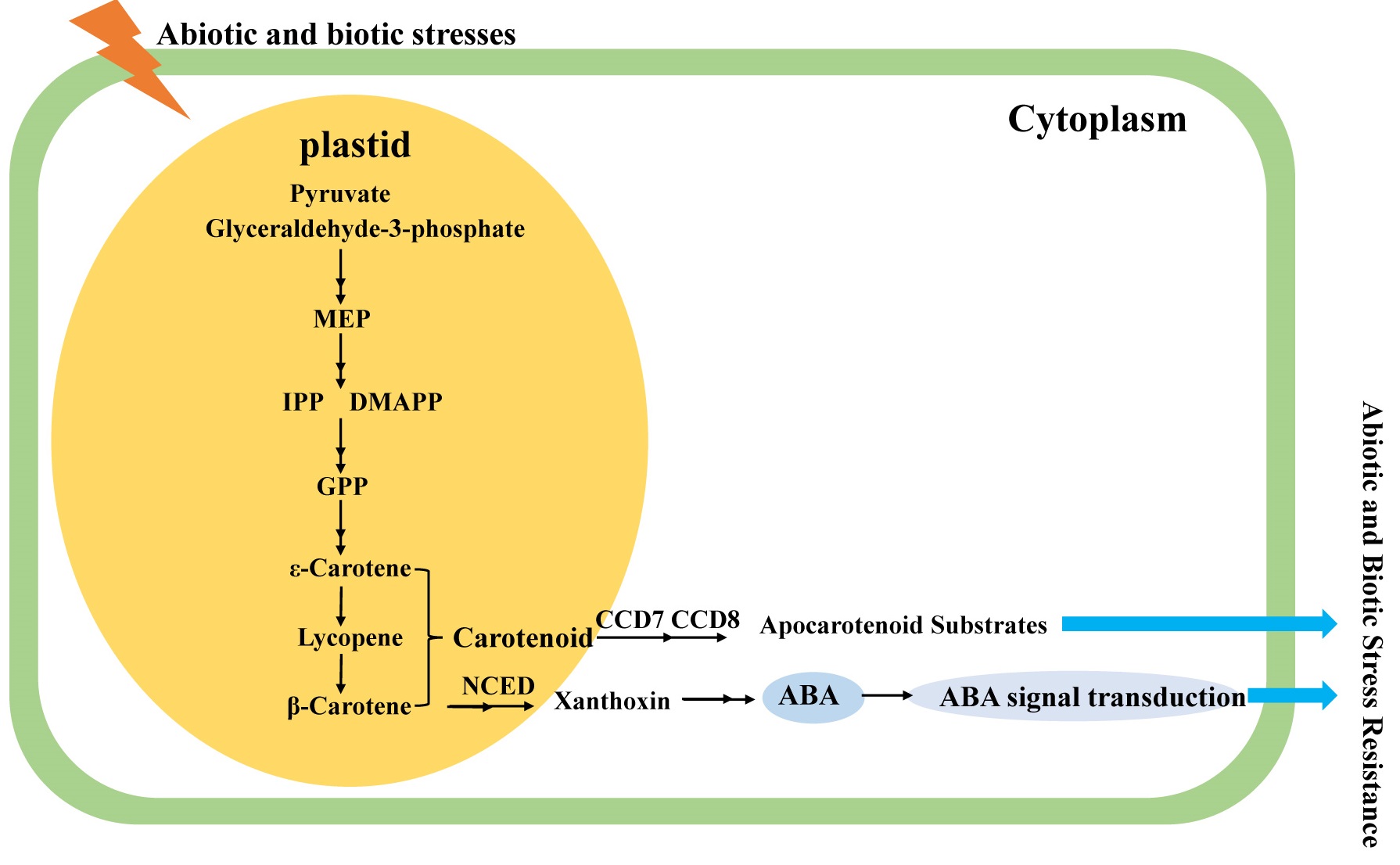

Supplement: Supplementary file 1 [file ijms-23-01418-s001.zip › Supplemental Figure s9.jpg]
